# Enhancing diagnostic deep learning via self-supervised pretraining on large-scale, unlabeled non-medical images

**DOI:** 10.1186/s41747-023-00411-3

**Published:** 2024-02-08

**Authors:** Soroosh Tayebi Arasteh, Leo Misera, Jakob Nikolas Kather, Daniel Truhn, Sven Nebelung

**Affiliations:** 1 Department of Diagnostic and Interventional Radiology, University Hospital RWTH Aachen, Aachen, Germany; 2 Institute and Polyclinic for Diagnostic and Interventional Radiology, Faculty of Medicine and University Hospital Carl Gustav Carus Dresden, Technische Universität Dresden, Dresden, Germany; 3 Else Kröner Fresenius Center for Digital Health, Technische Universität Dresden, Dresden, Germany; 4 Department of Medicine III, University Hospital RWTH Aachen, Aachen, Germany; 5 Medical Oncology, National Center for Tumor Diseases (NCT), University Hospital Heidelberg, Heidelberg, Germany

**Keywords:** Artificial intelligence, Deep learning, Medical image processing, Radiography (thoracic), Unsupervised machine learning

## Abstract

**Background:**

Pretraining labeled datasets, like ImageNet, have become a technical standard in advanced medical image analysis. However, the emergence of self-supervised learning (SSL), which leverages unlabeled data to learn robust features, presents an opportunity to bypass the intensive labeling process. In this study, we explored if SSL for pretraining on non-medical images can be applied to chest radiographs and how it compares to supervised pretraining on non-medical images and on medical images.

**Methods:**

We utilized a vision transformer and initialized its weights based on the following: (i) SSL pretraining on non-medical images (DINOv2), (ii) supervised learning (SL) pretraining on non-medical images (ImageNet dataset), and (iii) SL pretraining on chest radiographs from the MIMIC-CXR database, the largest labeled public dataset of chest radiographs to date. We tested our approach on over 800,000 chest radiographs from 6 large global datasets, diagnosing more than 20 different imaging findings. Performance was quantified using the area under the receiver operating characteristic curve and evaluated for statistical significance using bootstrapping.

**Results:**

SSL pretraining on non-medical images not only outperformed ImageNet-based pretraining (*p* < 0.001 for all datasets) but, in certain cases, also exceeded SL on the MIMIC-CXR dataset. Our findings suggest that selecting the right pretraining strategy, especially with SSL, can be pivotal for improving diagnostic accuracy of artificial intelligence in medical imaging.

**Conclusions:**

By demonstrating the promise of SSL in chest radiograph analysis, we underline a transformative shift towards more efficient and accurate AI models in medical imaging.

**Relevance statement:**

Self-supervised learning highlights a paradigm shift towards the enhancement of AI-driven accuracy and efficiency in medical imaging. Given its promise, the broader application of self-supervised learning in medical imaging calls for deeper exploration, particularly in contexts where comprehensive annotated datasets are limited.

**Graphical Abstract:**

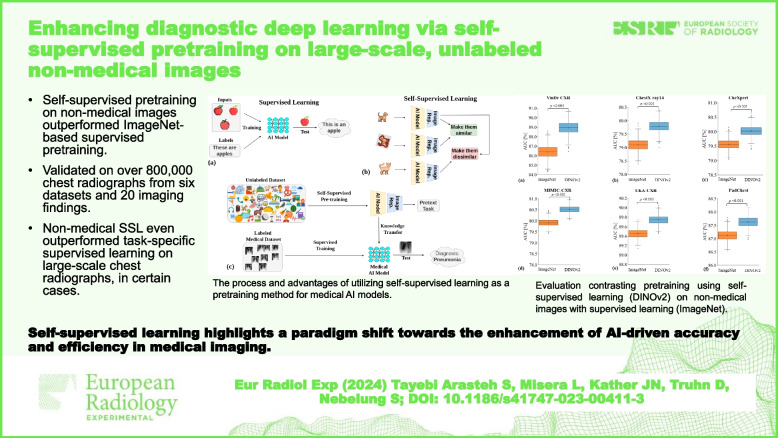

**Supplementary Information:**

The online version contains supplementary material available at 10.1186/s41747-023-00411-3.

## Key points

• Validated on over 800,000 chest radiographs from 6 datasets and 20 imaging findings, a self-supervised pretraining on non-medical images outperformed ImageNet-based supervised pretraining.

• Non-medical-self-supervised learning even outperformed task-specific supervised learning on large-scale chest radiographs, in certain cases.

• Self-supervised learning signifies AI’s transformative potential in medical imaging, especially chest radiography.

**Supplementary Information:**

The online version contains supplementary material available at 10.1186/s41747-023-00411-3.

## Background

Artificial intelligence (AI) has become an important tool in healthcare and medical image analysis [1]. Its application in radiology [2], specifically in automated diagnosis of chest radiographs [3], has gained increasing traction. Given the intricate challenges posed by the complexity and variability of chest radiographs, leveraging AI for improved interpretation is an important area of research and application. Since the number of labeled chest radiographs with definitive diagnosis available for the training of AI models is limited, interest in self-supervised learning (SSL) has grown.

SSL is a learning paradigm that allows models to derive rich representations from unlabeled data [4–6]. Unlike traditional supervised learning (SL), which relies on accurately labeled datasets that can be laborious and resource-intensive to create, SSL can be used with images only that contain no labels, offering a promising alternative for robust feature extraction. In addition, exciting possibilities arise from AI advancements, such as the evolution of transformer architectures from the realm of natural language processing (NLP) to computer vision [7]. The “vision transformer” (ViT), introduced in 2021 by Dosovitskiy et al. [8], replaces traditional convolution-based techniques with self-attention mechanisms [7], showing promise for healthcare applications. Nevertheless, further exploration is needed to fully integrate these advancements with existing pretraining methodologies [9], and we tackle this problem in our investigation.

It has been established in the literature that selecting an appropriate weight initialization for deep neural networks is a critical step that can influence the performance of AI models [10–12]. Usually, this is done by pretraining the network with SL on an unrelated task before training on the actual task. Numerous large-scale, public, annotated pretraining image datasets are available for this paradigm. The most widely used such datasets are ImageNet [13], the dataset of the Canadian Institute for Advanced Research, CIFAR [14] (CIFAR-10 and CIFAR-100), PASCAL Visual Object Classes [15], Microsoft Common Objects in Context [16], and places [17]. These datasets provide a valuable resource for initializing network weights when dedicated task-related pretraining weights are not accessible. In particular, the ImageNet database and its extended versions like ImageNet-21 K [13], trained on roughly 14 million annotated images, have enabled substantial performance increases of AI models and are widely regarded as the benchmark for pretraining deep learning models for image classification tasks [10–12].

**Fig. 1:**
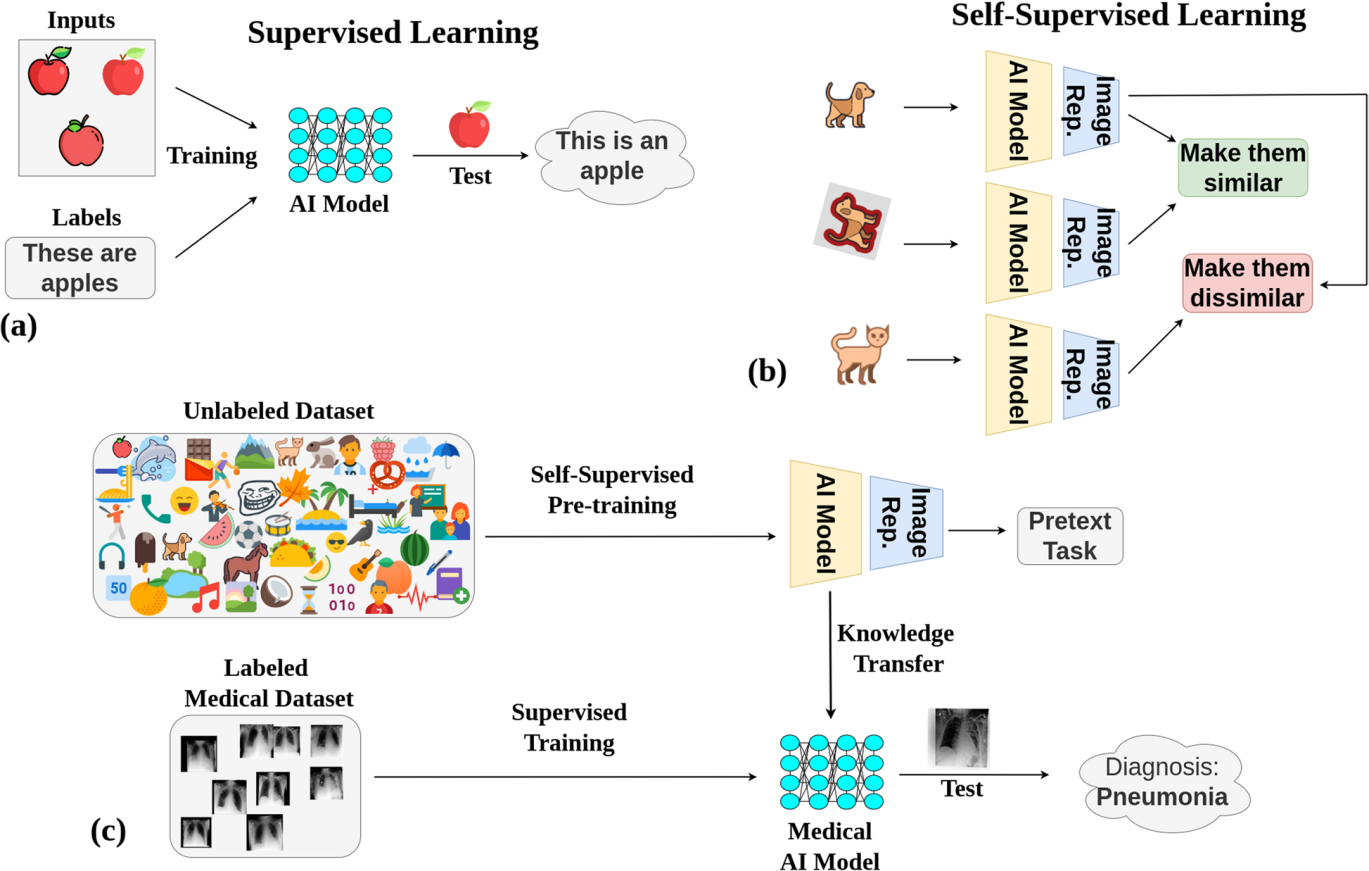
The process and advantages of utilizing self-supervised learning (SSL) as a pretraining method for medical AI models. **a** Supervised learning shows the traditional process of AI pretraining using labeled datasets, which can be resource- and time-intensive due to the need for manual annotation. **b** SSL paradigm where AI models are trained on unlabeled non-medical images, taking advantage of freely available data, bypassing the need for costly and time-consuming manual labeling. **c** Transfer of learnings from the SSL pretrained model using non-medical images to a supervised model for accurately diagnosing medical images, highlighting the potential for improved performance in medical AI models due to the large-scale knowledge gained from SSL

One drawback is that pretraining in this manner requires the images to be equipped with labels that depict what can be seen in the images. This naturally limits the number of available images, since labeling is a costly and resource-intensive procedure. Methods that use SSL, such as described in literature [4–6, 18–20], on the other hand have the advantage that images do not need to be labeled, and thus, much larger databases can be constructed (Fig. 1).

In this study, we investigate if pretraining with SSL on large unannotated image databases based on DINOv2 [18] can improve performance of medical AI models as compared to pretraining with SL. We examine this by training AI models to diagnose over 20 radiological imaging findings on an international multi-site dataset spanning three continents and comprising over 800,000 chest radiographs.

## Methods

### Patient cohorts

We analyzed frontal chest radiographs from six international patient cohorts across three continents, sourced from the VinDr-CXR [21], ChestX-ray14 [22], CheXpert [23], MIMIC-CXR [24], UKA-CXR [3, 25–28], and PadChest [29] datasets. Collectively, the study encompassed 805,805 radiographs from patients aged between 1 and 111 years. The median patient age was 61 years, with an average of 59 years and a standard deviation of 18 years. An overview of the characteristics for each dataset can be found in Table 1.

**Table 1:** Characteristics of the datasets utilized in this study

	VinDr-CXR	ChestX-ray14	CheXpert	MIMIC-CXR	UKA-CXR	PadChest
Number of radiographs (total)	18,000	112,120	157,878	213,921	193,361	110,525
Number of radiographs (training set)	15,000	86,524	128,356	170,153	153,537	88,480
Number of radiographs (test set)	3,000	25,596	29,320	43,768	39,824	22,045
Number of patients	N/A	30,805	65,240	65,379	54,176	67,213
Patient age (years) Median Mean ± standard deviation Range (minimum, maximum)	42 54 ± 18 (2, 91)	49 47 ± 17 (1, 96)	61 60 ± 18 (18, 91)	N/A N/A N/A	68 66 ± 15 (1, 111)	63 59 ± 20 (1, 105)
Patient’s sex Females/males [%] Training set, test set	47.8/52.2 44.1/55.9	42.4/57.6 41.9/58.1	41.4/58.6 39.0/61.0	N/A N/A	34.4/65.6 36.3/63.7	50.0/50.0 48.2/51.8
Projections [%] Anteroposterior Posteroanterior	0.0 100.0	40.0 60.0	84.5 15.5	58.2 41.8	100.0 0.0	17.1 82.9
Location	Hanoi, Vietnam	Maryland, USA	California, USA	Massachusetts, USA	Aachen, Germany	Alicante, Spain
Number of contributing hospitals	2	1	1	1	1	1
Labeling method	Manual	NLP (ChestX-ray14 labeler)	NLP (CheXpert labeler)	NLP (CheXpert labeler)	Manual	Manual & NLP (PadChest labeler)
Original labeling system	Binary	Binary	Certainty	Certainty	Severity	Binary
Accessibility of the dataset for research	Public	Public	Public	Public	Internal	Public

The table shows the statistics of the datasets used, including VinDr-CXR [21], ChestX-ray14 [22], CheXpert [23], MIMIC-CXR [24], UKA-CXR [3, 25–28], and PadChest [29]. The values correspond to only frontal chest radiographs, with the percentages of total radiographs provided. Binary labeling system refers to diagnosing if a finding is present or not. “Severity” refers to classification of the severity of a finding. “Certainty” indicates that a certainty level was assigned to each finding during the labeling by either the experienced radiologists (manual) or an automatic natural language processing—NPL, labeler. Note that some datasets may include multiple radiographs per patient

*N/A* Not available

### Label generation and parameters

This subsection delves into the label generation process, details the specific labels associated with each chest radiograph dataset, and references imaging parameters provided in the original studies. The labeled diseases within each dataset were not identical, but overlapped partially, details are given in Table 2.

**Table 2:** Distribution of different labels provided across datasets, considering only frontal images

Labels [n (%)]	VinDr-CXR	ChestX-ray14	CheXpert	MIMIC-CXR	UKA-CXR	PadChest
Atelectasis	148 (0.8%)	11,559 (10.3%)	26,313 (16.7%)	42,760 (19.9%)	-	6,166 (5.6%)
Atelectasis right	-	-	-	-	18,761 (9.7%)	-
Atelectasis left	-	-	-	-	15,082 (7.8%)	-
Calcification	371 (2.1%)	-	-	-	-	-
Cardiomegaly	2,126 (11.8%)	2,776 (2.5%)	19,890 (12.6%)	42,480 (19.7%)	90,348 (46.7%)	9,845 (8.9%)
Consolidation	217 (1.2%)	4,667 (4.2%)	9,542 (6.0%)	8,603 (4.0%)	-	1,666 (1.5%)
Edema	1 (0.0%)	2,303 (2.1%)	43,213 (27.4%)	24,663 (11.5%)	-	-
Emphysema	17 (0.1%)	2,516 (2.2%)	-	-	-	1,102 (1.0%)
Enlarged pulmonary artery	29 (0.2%)	-	-	-	-	-
Interstitial lung disease	373 (2.1%)	-	-	-	-	-
Lung opacity	631 (3.5%)	-	73,961 (46.8%)	40,876 (19.0%)	-	-
Lung cavity	29 (0.2%)	-	-	-	-	-
Lung cyst	6 (0.0%)	-	-	-	-	-
Lung lesion	-	-	5,829 (3.7%)	5,648 (2.6%)	-	-
Lung tumor	214 (1.2%)	-	-	-	-	-
Mediastinal shift	105 (0.6%)	-	-	-	-	-
Enlarged cardiomediastinum	-	-	7,787 (4.9%)	6,527 (3.0%)	-	-
Nodule/mass	585 (3.2%)	-	-	-	-	4,747 (4.3%)
Nodule	-	6,331 (5.6%)	-	-	-	-
Mass	-	5,782 (5.2%)	-	-	-	-
Pleural effusion	745 (4.1%)	13,317 (11.9%)	65,142 (41.3%)	48,716 (22.6%)	-	6,984 (6.3%)
Pleural effusion right	-	-	-	-	15,609 (8.1%)	-
Pleural effusion left	-	-	-	-	12,571 (6.5%)	-
Pleural thickening	1,051 (5.8%)	3,385 (3.0%)	-	-	-	3,372 (3.1%)
Pleural other	-	-	2,035 (1.3%)	1,751 (0.8%)	-	-
Pulmonary fibrosis	1,234 (6.9%)	1,686 (1.5%)	-	-	-	715 (0.6%)
Fracture	55 (0.3%)	-	6,445 (4.1%)	4,104 (1.9%)	-	-
COPD	9 (0.1%)	-	-	-	-	14,293 (12.9%)
Chronic changes	-	-	-	-	-	4,798 (4.3%)
Infiltrates	303 (1.7%)	19,894 (17.7%)	-	-	-	4,605 (4.2%)
Pneumonia	717 (4.0%)	1,431 (1.3%)	3,964 (2.5%)	13,916 (6.5%)	-	5,222 (4.7%)
Pneumonia right	-	-	-	-	22,513 (11.6%)	-
Pneumonia left	-	-	-	-	15,993 (8.3%)	-
Pneumothorax	76 (0.4%)	5,302 (4.7%)	16,277 (10.3%)	9,866 (4.6%)	-	-
Tuberculosis	646 (3.6%)	-	-	-	-	-
Scoliosis	-	-	-	-	-	5,573 (5.0%)
Hernia	-	227 (0.2%)	-	-	-	1,609 (1.5%)
Congestion	-	-	-	-	16,371 (8.5%)	863 (0.8%)
Support devices	-	-	90,967 (57.6%)	61,358 (28.5%)	-	-
Aortic enlargement	2,566 (14.3%)	-	-	-	-	-
Aortic elongation	-	-	-	-	-	8,116 (7.3%)
Kyphosis	-	-	-	-	-	2,621 (2.4%)
Sternotomy	-	-	-	-	-	1,912 (1.7%)
Cavitation	-	-	-	-	-	353 (0.3%)
Volume loss	-	-	-	-	-	1,647 (1.5%)
Pacemaker	-	-	-	-	-	2,294 (2.1%)
Bronchiectasis	-	-	-	-	-	1,548 (1.4%)
Air trapping	-	-	-	-	-	3,471 (3.1%)
No finding (healthy)	12,652 (70.3%)	60,361 (53.8%)	17,000 (10.8%)	81,117 (37.7%)	74,455 (38.5%)	36,148 (32.7%)

The values indicate the total certain positive cases within an entire dataset. UKA-CXR specifies separate labels for the presence of atelectasis, pleural effusion, and pneumonia on both the right and left chest sides

#### VinDr-CXR

The VinDr-CXR [21] dataset, collected between 2018 and 2020, sourced over 100,000 chest radiographs from two Vietnamese hospitals’ picture archiving and communication system servers. These images were captured using a broad spectrum of scanners from different medical equipment brands. The dataset was carefully anonymized for patient privacy. A Python script removed digital imaging and communications in medicine (DICOM) tags with protected health information (PHI) [30], keeping only vital image processing attributes. Textual data on the images was auto erased, with a manual check ensuring no text remained. While the primary focus was on adult posteroanterior-view chest radiographs, the collection did have outliers, which were filtered using a binary classifier. The dataset was annotated for 28 findings and diagnoses, including 22 localized and 6 global labels. Expert radiologists curated these labels based on condition prevalence and visibility in chest radiographs. Using a web-based system [31], 17 radiologists labeled the data. From the refined data, 18,000 radiographs were selected, with 15,000 designated for training and 3,000 for testing. Three radiologists independently annotated each image, and for the test set, any disagreements were resolved by two senior radiologists to ensure label accuracy [21].

#### ChextX-ray14

The ChestX-ray14 [22] dataset targets fourteen common thoracic pathologies, identified through radiologists’ input. Using these pathologies as keywords, related radiological reports and images were extracted from the picture archiving and communication system. Through NLP techniques [32], reports were labeled based on the presence or absence of the specified pathologies while also excluding negations and uncertainties. The labeling process involved two main steps [22]: (i) initially detecting disease concepts primarily from report sections and then (ii) categorizing undetected reports as “normal.” Disease identification was enhanced using DNorm [33] and MetaMap [34]. To ensure accurate labeling, the team integrated advanced methodologies for handling negations and uncertainties, leveraging tools like NLTK [35], the Bllip parser [36], David McClosky’s biomedical model [37], and the Stanford dependencies converter [38]. A “normal” label was applied if no disease was detected or if the report indicated normalcy. The labeling approach’s accuracy was validated using the OpenI API [39, 40].

#### CheXpert

The CheXpert [23] dataset includes 224,316 frontal and lateral chest radiographs from 65,240 patients, collected from Stanford Hospital between 2002 and 2017. Each radiograph is annotated for 14 clinically relevant observations [41] as positive, negative, or uncertain. The selection of these observations emerged from the manual review of 1,000 associated radiology reports by a board-certified radiologist. The labeling process hinged on a rule-based NLP labeler and transpired in three stages. Key observations were gleaned from the Impression section of the radiology reports. This extraction used a comprehensive list of phrases, meticulously curated by radiologists. The subsequent phase saw these extracted mentions being classified as negative, uncertain, or positive. Any ambiguities in the report, or direct expressions of uncertainty by the radiologist, were categorized as “uncertain.” If a mention was not distinctly categorized, it defaulted to a positive label. Following a procedure similar to NegBio [42], this classification leaned on tools such as NLTK [35], the Bllip parser [36], and Stanford CoreNLP [43], seeking a universal dependency parse of the report. Finally, the individual mention classifications coalesced to assign a conclusive label to each of the 14 observations. The absence of a mention was labeled as blank [23].

#### MIMIC-CXR

The MIMIC-CXR [24] dataset encompasses 377,110 frontal and lateral images stemming from 227,835 radiographic studies conducted at Beth Israel Deaconess Medical Center, Boston, MA, USA. Chest radiographs from 2011 to 2016 were identified, and all corresponding reports within this timeframe were extracted. The radiographs, sourced in DICOM format, faced rigorous de-identification processes, particularly for potential PHI in meta-data and “burned in” annotations [24]. Further, the reports underwent a detailed, rule-based de-identification, producing two primary segments: an optional addendum and the primary report body—both penned by radiologists. Extraneous details were trimmed, and any PHI was uniformly replaced with underscores. Notably, the same NLP labeler employed in the CheXpert [23] dataset was applied to these reports. This facilitated the automatic generation of labels for the chest radiographs, categorizing the 14 imaging findings, consistent with CheXpert, as positive, negative, or uncertain. To validate the de-identification process, 2,238 radiology reports were manually annotated to detect PHI. This manual process identified eight tokens of PHI that the automated method overlooked, which were subsequently removed [24].

#### UKA-CXR

The UKA-CXR [3, 25–28], an internal dataset from University Hospital RWTH Aachen, Germany, includes frontal chest radiographs collected between 2009 and 2020. Captured across 10 varied intensive care units using 18 distinct mobile radiography systems by over 70 specialized radiologic technologists, the methodology evolved from conventional screen-film systems to digital flat-panel detectors by 2016. Despite diverse patient positioning and source-to-digital film distances, all images were consistently shot in the anteroposterior orientation, facilitated by automatic exposure control. Labeling involved a rigorous review of each radiograph by one of 98 radiologists on designated clinical workstations, employing a standardized template. These radiologists, accredited or guided by board-certified colleagues, adhered to established radiologic conventions while evaluating the images [3]. The dataset features labels like pleural effusion, pneumonia, atelectasis, congestion, and cardiomegaly, each segmented into five distinct severity or extent gradations. For instance, cardiomegaly ranged from “normal” to “massively enlarged,” whereas other labels spanned classifications such as “negative,” “mild,” “moderate,” “severe,” and “uncertain mild” [3, 25].

#### PadChest

The PadChest [29] dataset, derived from the Hospital Universitario de San Juan in Alicante, Spain, encompasses studies from 2009 to 2017, totaling 109,931 studies and 168,861 distinct frontal and lateral images. All data was de-identified. The images were dynamically rescaled based on DICOM parameters, with no resizing to maintain resolution. Projection and body position information were used to categorize images into six primary groups: standard posteroanterior, standard lateral, anteroposterior vertical, anteroposterior horizontal, pediatric, and rib views [29]; 27% of the reports, which translates to 27,593 studies, were manually annotated by radiologists. This was streamlined by an automated topic extraction process, which presented radiologists with frequently occurring sentences, allowing for more efficient and consistent labeling. Once this subset of data was labeled, it was used to train a multilabel text classifier which was then employed to automatically annotate the remaining 73% of the reports [29].

### Experimental design

**Fig. 2:**
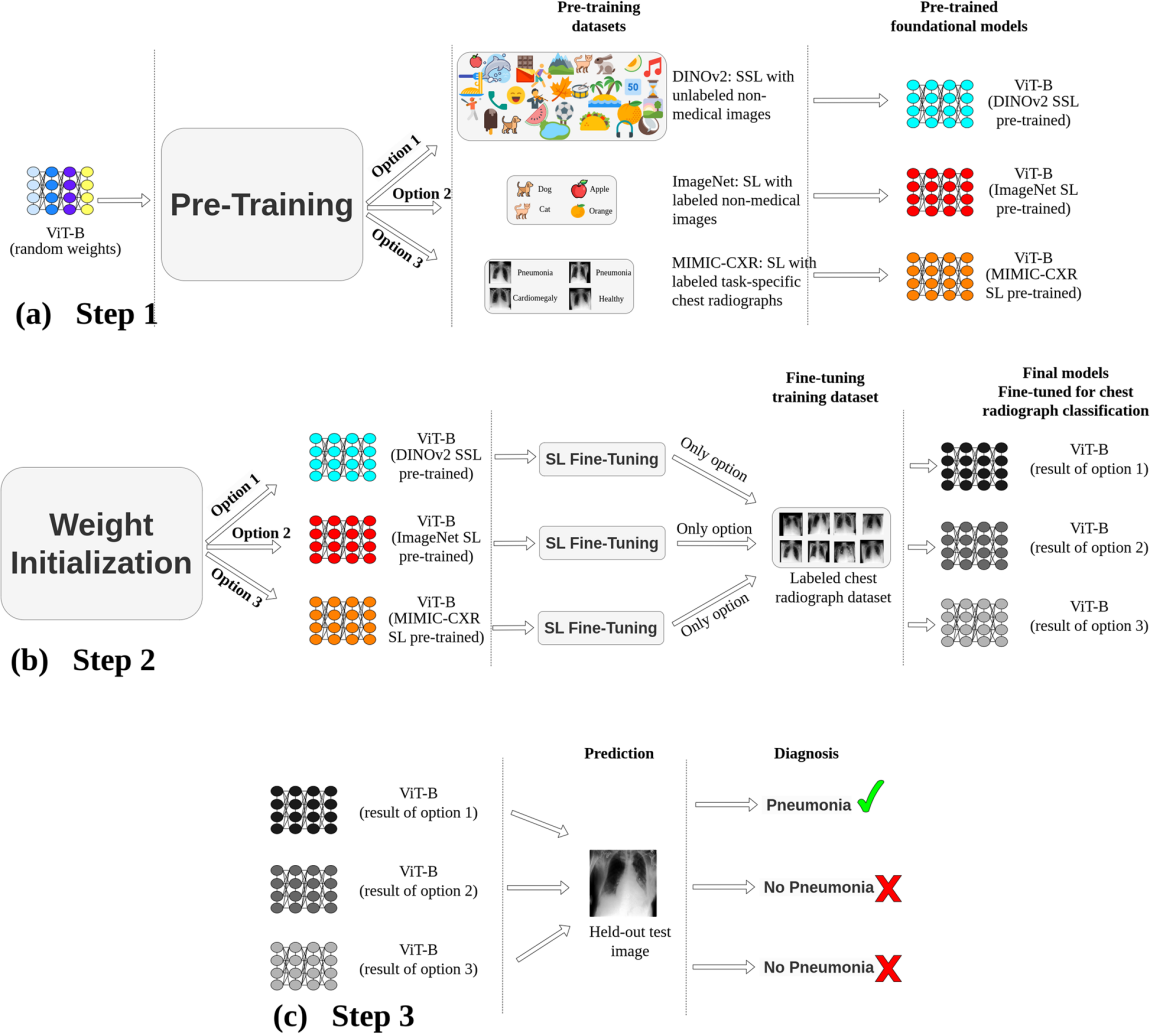
General methodology. **a** Pretraining: the vision transformer base (ViT-B) undergoes pretraining through three avenues: (i) self-supervised learning (SSL) on non-medical images (DINOv2(18)), (ii) supervised learning (SL) using ImageNet-21 K [13], and (iii) SL based on MIMIC-CXR [24] chest radiographs. **b** ViT-B models are subsequently fine-tuned using labeled chest radiographs from various datasets. **c** Prediction: diagnostic performance of these models is assessed using images from unseen test sets from various datasets. Although this figure exemplifies pneumonia prediction using a single dataset, steps 2 (fine-tuning) and 3 (systematic evaluation) were consistently implemented across six major datasets: VinDr-CXR (*n* = 15,000 training, *n* = 3,000 testing), ChestX-ray14 (*n* = 86,524 training, *n* = 25,596 testing), CheXpert (*n* = 128,356 training, *n* = 39,824 testing), MIMIC-CXR (*n* = 170,153 training, *n* = 43,768 testing), UKA-CXR (*n* = 153,537 training, *n* = 39,824 testing), and PadChest (*n* = 88,480 training, *n* = 22,045 testing). The refined models identify a total of 22 distinct imaging findings

A schematic representation of the study methodology is presented in Fig. 2. The process commenced with step 1, *i.e.*, the pretraining of a ViT [8] base model. This was achieved through three distinct strategies: (i) SSL with non-medical images, DINOv2 [18], (ii) SL on ImageNet-21 K [13], and (iii) SL with MIMIC-CXR chest radiographs [24]. Step 2 involved fine-tuning the models using labeled chest radiographs. Finally, in step 3, the refined models underwent an evaluation process, where they were tested using images from held-out test sets of chest radiographs from different domains.

### Network architecture

Our study employed the original 12-layer vision transformer (ViT) base (ViT-B) model as devised by Dosovitskiy et al. [8]. This network ingested image inputs of dimensions (224 × 224 × 3) in batches of 32. For compatibility with the red, green, and blue (RGB) format of pretraining images, grayscale radiographs were replicated across three channels while retaining their grayscale nature. The embedding layer featured dimensions of either (16 × 16) or (14 × 14), depending on the pretrained weights available. A convolution operation with strides of (16 × 16) or (14 × 14) ensued, followed by a positional embedding layer. This sequence generated an output sequence of vectors featuring a hidden layer size of 768. These vectors were subsequently inputted to a standard transformer encoder. A fully connected layer constituted the classification head, employing a binary sigmoid function to convert the output predictions into individual class probabilities.

#### Step 1: pretraining

##### SSL pretraining on non-medical images (DINOv2)

DINOv2 [18], an advancement of the DINO [44] method by Meta AI, focuses on self-supervised learning, striving to extract diverse visual features from a vast, curated dataset. Initially comprising 1.2 billion images drawn from a variety of online sources, the dataset went through a rigorous deduplication process [45, 46], culminating in the refined LVD-142 M [18] dataset with 142 million unique images. This curation integrated images from notable datasets like ImageNet, Google Landmarks, and an array of broader public and internal web repositories. Using embeddings from the “Huge” iteration of the ViT network architecture (ViT-H) [8] pretrained on ImageNet [13], a connection was established between curated and uncurated images, paving the way for the LVD-142 M dataset. From this foundation, several ViT models, aligned with the DINOv2 training methodology, were developed. The ViT base (ViT-B) [8] iteration of this model served as the weight reference for our study.

The essence of DINOv2 synthesizes elements from DINO [44] and iBOT [47] losses, enhanced by the centering technique of SwAV [48]. The approach incorporates dual primary objectives: image level and patch level. The image-level objective deploys a cross-entropy loss between features extracted from varying crops of an identical image using a ViT, from both a student and a teacher network built with an exponential moving average of past iterates [49]. In contrast, the patch-level objective operates by selectively masking certain input patches for the student, followed by the application of a cross-entropy loss between the patch features of both the student and teacher networks [47]. To combat issues of overfitting and underfitting, the weights associated with these objectives were decoupled. To ensure uniform feature distribution, the Sinkhorn-Knopp [50] normalization and the KoLeo regularizer [51] were employed [48, 52]. While models trained at a 416 × 416 resolution showcased optimal performance across various resolutions, they necessitated nearly triple the computational capacity compared to the 224 × 224 resolution. Nonetheless, a balanced approach was adopted by conducting self-supervised training at 224 × 224 and amplifying the resolution only in the concluding iterations, delivering near-optimal results without an exorbitant computational burden [53]. For more detailed information regarding data preparation, training, and optimization steps, please refer to the original paper [18].

##### SL pretraining on non-medical images (ImageNet)

ImageNet [13] is a vast database with diverse, annotated non-medical images. The subset, ImageNet-21 K, houses over 14 million images of various resolutions across 21,841 categories. Using supervised learning (SL), a ViT-B model (patch size 16 × 16, input size 224 × 224 × 3) was trained end to end on the complete ImageNet-21 K to predict among the 21,841 available categories.

##### SL pretraining on chest radiographs (MIMIC-CXR)

MIMIC-CXR [24] stands as the largest public chest radiograph dataset to date. Adopting a training approach similar to that of ImageNet [13], a ViT-B model was trained on MIMIC-CXR for classifying specific imaging findings relevant to our fine-tuning datasets. Unlike the foundational models established using DINOv2 [18] and ImageNet, this strategy directly targets the specific task at hand. Despite the smaller dataset size compared to the prior two methods, the task-specific nature and substantial scale of MIMIC-CXR suggest potential for enhanced performance at first glance.

#### Step 2: fine-tuning (SL training on chest radiographs)

##### Choice of the training chest radiographs for fine-tuning

For benchmarking, six chest radiograph datasets were standardized using only frontal images for both fine-tuning and evaluation. Original sets from VinDr-CXR and ChestX-ray14 were retained, while CheXpert, MIMIC-CXR, UKA-CXR, and PadChest were divided into 80% training and 20% test sets based on patients. This ensured radiographs from one patient stayed together, preserving patient-specific integrity. Training sets had 128, 356, 170, 153, 153, 537, and 88,480 images for CheXpert, MIMIC-CXR, UKA-CXR, and PadChest, respectively. Test sets contained 29, 320, 43, 768, 39, 824, and 22,045 images correspondingly. Consistent sets were used across all steps for comparable evaluations [25–27].

##### Label unification

In line with previous studies [25, 26, 28], a binary multilabel classification approach was employed, permitting each image to receive a positive or negative diagnosis for each disease. Optimization was centered on the average performance across all labels, without delving into detailed comparisons for individual diseases. For datasets with certainty levels (CheXpert and MIMIC-CXR), labels were converted to binary: classifications marked as “certain negative” and “uncertain” were categorized as negative, while “certain positive” was deemed positive. The final breakdown of the labels employed for each dataset’s multilabel diagnosis in this study is provided in Table 3. Labels with minimal representation were excluded from our final label selection, *e.g.*, “lung cyst” and “edema” in the VinDr-CXR dataset had only 6 and 1 positive instances, respectively (refer to Table 2). Thus, they were excluded from our final label selection for the VinDr-CXR dataset (see Table 3).

**Table 3:** Breakdown of labels used for multilabel diagnosis across datasets in this study

Labels	VinDr-CXR	ChestX-ray14	CheXpert	MIMIC-CXR	UKA-CXR	PadChest
Cardiomegaly	✔	✔	✔	✔	✔	✔
Pleural effusion	✔	✔	✔	✔		✔
Pleural effusion right					✔	
Pleural effusion left					✔	
Pleural thickening	✔	✔				✔
Infiltrates						✔
Pneumonia	✔	✔	✔	✔		✔
Pneumonia right					✔	
Pneumonia left					✔	
Pneumothorax	✔	✔	✔	✔		✔
Atelectasis	✔	✔	✔	✔		✔
Atelectasis right					✔	
Atelectasis left					✔	
Consolidation	✔	✔	✔	✔		✔
Congestion					✔	✔
Nodule/mass	✔					✔
Nodule		✔				
Mass		✔				
Fibrosis	✔	✔				
Hernia		✔				✔
Emphysema		✔				✔
Edema		✔				
Aortic elongation						✔
Kyphosis						✔
COPD						✔
Scoliosis						✔
Lung opacity	✔		✔	✔		
Lung lesion			✔	✔		
Fracture			✔	✔		
No finding (healthy)	✔	✔	✔	✔	✔	✔

The table details the specific labels applied to each dataset’s images for diagnostic purposes. The study’s multilabel diagnosis tasks involved predicting 11, 14, 10, 10, 9, and 17 distinct labels for the VinDr-CXR, ChestX-ray14, CheXpert, MIMIC-CXR, UKA-CXR, and PadChest datasets, respectively. Notably, UKA-CXR delineates separate labels for the presence of atelectasis, pleural effusion, and pneumonia for both the right and left sides of the chest. The “Healthy” label signifies cases without any disease diagnosis. *✔* label utilized in this study

*COPD* Chronic obstructive pulmonary disease

Overall, our analysis encompassed 30 labels spanning all datasets. The specific number of these labels within the VinDr-CXR, ChestX-ray14, CheXpert, MIMIC-CXR, UKA-CXR, and PadChest datasets was 11, 14, 10, 10, 9, and 17, respectively. A detailed breakdown of these labels per dataset can be found in Table 3.

##### Standardized image preprocessing

To standardize and ensure equitable comparisons across various SL fine-tuning experiments, we uniformly applied a consistent image preprocessing approach to all chest radiograph datasets for fine-tuning. This preprocessing sequence began with resizing all images to a consistent dimension of 224 × 224 pixels. Subsequently, min–max feature scaling, as suggested by Johnson et al. [24], was employed. Finally, to enhance image contrast and thereby aid in more accurate disease identification, we applied histogram equalization to the processed images [25–27].

##### SL training configuration

All ViT models were optimized using the AdamW [54] optimizer with learning rates set at 1 × 10^-5^. The network comprised approximately 86 million trainable parameters. Data augmentation strategies included random rotation within the range of [0, 8] degrees and random flipping [25]. Each network was trained end to end, *i.e.*, optimizing all the parameters, in a supervised learning manner employing each of the three sets of pretrained weights as initial weights.

It is noteworthy that class imbalance is a pervasive issue in numerous medical image datasets, often resulting in biased model training that disproportionately favors the majority class [55]. This is evidenced in our study by Table 2, which presents the distribution of positive labels for each dataset, revealing distinct variations in distributions. To address this concern, binary weighted cross-entropy [56], a modification of the standard binary cross-entropy, was utilized as our loss function. Weights for individual labels were determined based on the inverse frequency of each label within the training data for the respective dataset [3, 25–27].

#### Step 3: evaluation and statistical analysis

Test sets, held out from the training sets of each dataset, remained consistent across all experiments for benchmarking. The primary evaluation metric for our study was the area under the receiver operating characteristic curve (ROC-AUC), supported by accuracy, specificity, and sensitivity, calculated with a threshold that was determined according to the Youden’s criterion [57]. We employed bootstrapping [58] with replacement, on each test set with 1,000 redraws for each ROC-AUC value to determine the statistical spread in terms of mean ± standard deviation and to calculate *p*-values. Multiplicity-adjusted *p*-values were determined based on the false discovery rate to account for multiple comparisons, and the family-wise alpha threshold was set at 0.050.

## Results

### Pretraining with SSL *versus* SL for medical AI models

**Fig. 3:**
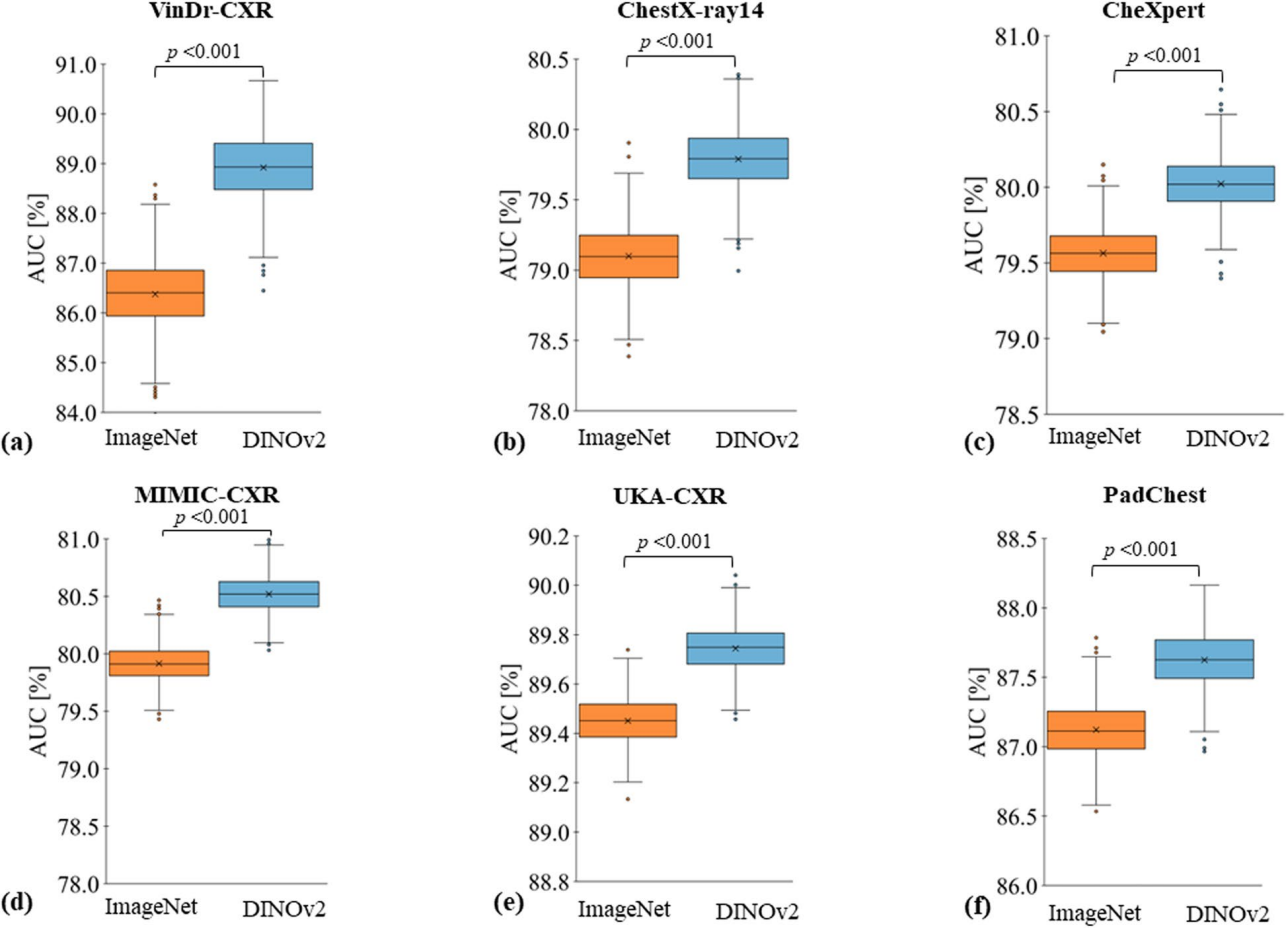
Evaluation contrasting pretraining using self-supervised learning (SSL) on non-medical images with supervised learning (SL). Models were either pretrained with SSL (DINOv2, shown in blue) or with SL (ImageNet [13], shown in orange) on non-medical, non-medical images. Subsequently, these models were fine-tuned on chest radiographs in a supervised manner for six datasets: (**a**) VinDr-CXR [21], (**b**) ChestX-ray14 [22], (**c**) CheXpert [23], (**d**) MIMIC-CXR [24], (**e**) UKA-CXR [3, 25–28], and (**f**) PadChest [29] with fine-tuning training images of *n* = 15,000, *n* = 86,524, *n* = 128,356, *n* = 170,153, *n* = 153,537, and *n* = 88,480, respectively, and test images of *n* = 3,000, *n* = 25,596, *n* = 39,824, *n* = 43,768, *n* = 39,824, and *n* = 22,045, respectively. The box plots present the mean area under receiver operating characteristic curve (ROC-AUC) values across all labels within each dataset. A consistent pattern emerges, showing SSL-trained models outperforming SL pretrained ones. Crosses denote means; boxes define the interquartile range (from Q1 to Q3), with the central line signifying the median (Q2). Whiskers stretch to 1.5 times the interquartile range above Q3 and below Q1. Points beyond this range are marked as outliers. Statistical differences between the DINOv2 and ImageNet approaches were evaluated through bootstrapping, with corresponding *p*-values displayed. Note the varying *y*-axis scales

**Fig. 4:**
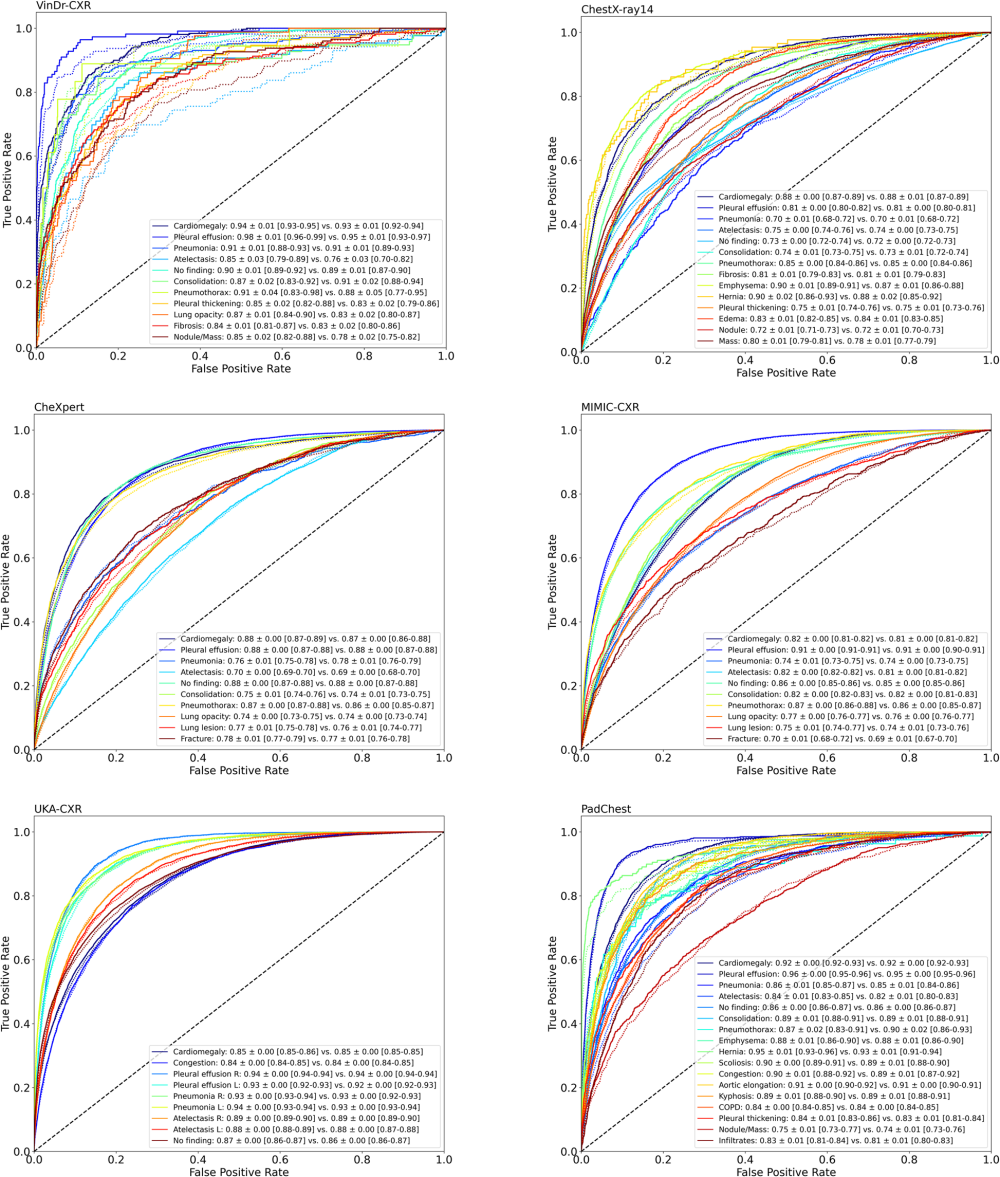
Receiver operating characteristic (ROC) curves of individual labels comparing diagnostic models pretrained with self-supervised learning (SSL) on non-medical images against fully supervised learning (SL) on non-medical images. Models pretrained via SSL used DINOv2 (solid lines), while SL utilized ImageNet (dotted lines). These models were subsequently fine-tuned in a supervised manner on chest radiographs from six datasets: VinDr-CXR, ChestX-ray14, CheXpert, MIMIC-CXR, UKA-CXR, and PadChest. The number of training images for SL fine-tuning for each dataset was *n* = 15,000, *n* = 86,524, *n* = 128,356, *n* = 170,153, *n* = 153,537, and *n* = 88,480, and test images were *n* = 3,000, *n* = 25,596, *n* = 39,824, *n* = 43,768, *n* = 39,824, and *n* = 22,045, respectively. Corresponding area under ROC curve values for each label, presented as mean ± standard deviation (95% CI), is provided in the bottom right, contrasting DINOv2 *versus* ImageNet pretraining strategies

We compare two settings for the pretraining stage of AI models: in the first setting, pretraining is performed using SSL on the DINOv2 [18] dataset; in the second setting, pretraining is done with SL on ImageNet-21 K [13]. For both settings, we subsequently fine-tune the AI model on radiographs to classify the presence of a disease. We consistently observe superior classification performance for the first setting. The models that were pretrained with SSL exhibit significantly superior performance in terms of the average over all ROC-AUC values for individual labels as compared to those pretrained with SL for all datasets (VinDr-CXR 88.92 ± 4.59% [mean ± standard deviation] *versus* 86.38 ± 6.27%; ChestX-ray14 79.79 ± 6.55% *versus* 79.10 ± 6.34%; CheXpert 80.02 ± 6.60% *versus* 79.56 ± 6.51%; MIMIC-CXR 80.52 ± 6.17% *versus* 79.92 ± 6.35%; UKA-CXR 89.74 ± 3.57% *versus* 89.45 ± 3.62%; and PadChest: 87.62 ± 4.86% *versus* 87.12 ± 5.05%; *p* < 0.001 for all dataset pairs). Figures 3 and 4 display the receiver operating characteristic curves for all individual labels, encompassing a total of 30 unique labels, which consist of 22 specific imaging findings and healthy participants, across each dataset for both methodologies. Table 3 provides a detailed breakdown of the classification targets for each dataset, and Table 4 provides a comprehensive comparison of the average ROC-AUC, accuracy, sensitivity, and specificity for each fine-tuning dataset. For an even more detailed comparison, Supplementary Tables S1–S6 provide individual evaluation metrics for each label.

**Table 4:** Comparative evaluation of pretraining with self-supervision on non-medical images *versus* full supervision on non-medical images

	Pretraining	VinDr-CXR	ChestX-ray14	CheXpert	MIMIC-CXR	UKA-CXR	PadChest
ROC-AUC	DINOv2	88.92 ± 4.59	79.79 ± 6.55	80.02 ± 6.60	80.52 ± 6.17	89.74 ± 3.57	87.62 ± 4.86
	ImageNet-21 K	86.38 ± 6.27	79.10 ± 6.34	79.56 ± 6.51	79.92 ± 6.35	89.45 ± 3.62	87.12 ± 5.05
Accuracy	DINOv2	82.49 ± 6.92	72.81 ± 7.43	72.37 ± 8.29	73.08 ± 5.32	80.68 ± 4.00	79.82 ± 6.69
	ImageNet-21 K	81.92 ± 6.50	71.69 ± 7.29	71.36 ± 8.39	73.00 ± 5.37	79.94 ± 4.29	78.73 ± 7.49
Sensitivity	DINOv2	83.58 ± 6.93	73.14 ± 8.94	75.68 ± 6.45	74.87 ± 10.01	83.42 ± 4.57	81.66 ± 6.91
	ImageNet-21 K	78.50 ± 8.97	73.04 ± 8.23	75.43 ± 6.00	73.91 ± 9.51	83.76 ± 4.37	81.80 ± 5.30
Specificity	DINOv2	81.69 ± 7.37	73.32 ± 8.00	70.95 ± 9.69	72.25 ± 6.04	80.32 ± 4.44	79.49 ± 6.97
	ImageNet-21 K	81.80 ± 6.88	72.10 ± 7.94	70.23 ± 9.33	72.30 ± 6.16	79.39 ± 4.61	78.37 ± 7.80
ROC-AUC *p*-value		0.001	0.001	0.001	0.001	0.001	0.001

The metrics used for comparison include the area under the receiver operating characteristic curve (ROC-AUC), accuracy, sensitivity, and specificity percentage values, all averaged over all labels for each dataset. The datasets in question are those pretrained with self-supervision on non-medical images (DINOv2 [18]) and those under full supervision with non-medical images (ImageNet-21 K [13]). The datasets employed in this study are VinDr-CXR, ChestX-ray14, CheXpert, MIMIC-CXR, UKA-CXR, and PadChest, with fine-tuning training images totals of *n* = 15,000, *n* = 86,524, *n* = 128,356, *n* = 170,153, *n* = 153,537, and *n* = 88,480, respectively, and test images totals of *n* = 3,000, *n* = 25,596, *n* = 39,824, *n* = 43,768, *n* = 39,824, and *n* = 22,045, respectively. For more information on the different labels used for each dataset, please refer to Table 3. *p*-values are given for the comparison between the ROC-AUC results obtained from DINOv2 and ImageNet-21 K pretraining weights

### SSL pretraining on non-medical images *versus* SL pretraining on radiographs

In the preceding experiment, we investigated pretraining using SSL and SL on non-medical images. An alternative approach to such pretraining on unrelated tasks is pretraining on medical images, potentially even with SL if labels are available. Here, we compare two settings: (i) pretraining with SSL on non-medical images (as before) *versus* (ii) pretraining with SL on 210,625 radiographs from the MIMIC-CXR [24] dataset. This dataset is currently the most comprehensive dataset of chest radiographs that is publicly available. We pretrained the network on this dataset by aligning all labels from the MIMIC-CXR dataset with each of the other datasets respectively, selecting all overlapping labels. This led to the identification of up to 10 different imaging findings for each dataset.

For both scenarios, we then trained networks for the task at hand, *i.e.*, for classification in VinDr-CXR, ChestX-ray14, CheXpert, UKA-CXR, and PadChest. Table 5 presents the ROC-AUC values for individual labels for each dataset. We find that for large datasets, approach (i) performs better CheXpert (ROC-AUC 80.02 ± 6.60% [mean ± standard deviation] *versus* 79.45 ± 6.60%, *p* < 0.001) and UKA-CXR (ROC-AUC 88.49 ± 2.65% *versus* 88.32 ± 2.77%, *p* = 0.001). However, for small datasets, approach (ii) performs better VinDr-CXR (ROC-AUC = 91.58 ± 3.45% *versus* 94.47 ± 3.30%, *p* < 0.001); ChestX-ray14 (ROC-AUC 77.99 ± 6.38% *versus* 78.68 ± 6.77%, *p* < 0.001); and PadChest (ROC-AUC 87.89 ± 4.30% *versus* 89.30 ± 4.45%, *p* < 0.001).

**Table 5:** Comparison of pretrained weights: self-supervised learning with large non-medical images *versus* supervised learning with a large, task-specific chest radiograph dataset

Labels	VinDr-CXR		ChestX-ray14		CheXpert		UKA-CXR		PadChest	
	DINOv2	MIMIC-CXR	DINOv2	MIMIC-CXR	DINOv2	MIMIC-CXR	DINOv2	MIMIC-CXR	DINOv2	MIMIC-CXR
Cardiomegaly	94.53 ± 0.52	97.17 ± 0.34	88.51 ± 0.47	89.54 ± 0.44	87.96 ± 0.31	87.27 ± 0.31	85.86 ± 0.18	85.45 ± 0.18	92.30 ± 0.27	92.68 ± 0.26
Pleural effusion	97.62 ± 0.68	98.31 ± 0.52	81.01 ± 0.32	82.00 ± 0.32	87.81 ± 0.20	87.64 ± 0.20	91.23 ± 0.19	91.41 ± 0.19	95.66 ± 0.26	95.85 ± 0.24
Pneumonia	91.99 ± 0.98	94.46 ± 0.66	70.17 ± 1.03	69.85 ± 1.04	76.42 ± 0.88	76.29 ± 0.84	92.15 ± 0.18	91.94 ± 0.18	83.93 ± 0.67	84.96 ± 0.66
Atelectasis	88.55 ± 1.71	92.21 ± 1.48	75.56 ± 0.43	75.87 ± 0.41	69.57 ± 0.40	69.28 ± 0.39	86.36 ± 0.23	86.30 ± 0.24	83.62 ± 0.58	83.59 ± 0.55
Consolidation	91.35 ± 1.56	94.82 ± 0.74	73.60 ± 0.57	75.11 ± 0.54	75.14 ± 0.56	74.13 ± 0.56	N/A	N/A	88.26 ± 0.82	89.95 ± 0.76
Pneumothorax	90.96 ± 2.91	97.39 ± 1.27	84.70 ± 0.38	85.93 ± 0.37	87.29 ± 0.33	86.03 ± 0.34	N/A	N/A	86.37 ± 2.01	92.89 ± 1.00
Lung opacity	86.86 ± 1.27	87.89 ± 1.26	N/A	N/A	73.98 ± 0.28	73.62 ± 0.29	N/A	N/A	N/A	N/A
Lung lesion	N/A	N/A	N/A	N/A	76.56 ± 0.73	75.79 ± 0.73	N/A	N/A	N/A	N/A
Fracture	N/A	N/A	N/A	N/A	77.93 ± 0.67	76.92 ± 0.66	N/A	N/A	N/A	N/A
No finding (healthy)	90.79 ± 0.56	93.51 ± 0.46	72.37 ± 0.33	72.48 ± 0.33	87.61 ± 0.30	87.53 ± 0.31	86.86 ± 0.18	86.49 ± 0.18	85.11 ± 0.26	85.20 ± 0.26
*Average*	*91.58* ± *3.45*	*94.47* ± *3.30*	*77.99* ± *6.38*	*78.68* ± *6.77*	*80.03* ± *6.60*	*79.45* ± *6.60*	*88.49* ± *2.65*	*88.32* ± *2.77*	*87.89* ± *4.30*	*89.30* ± *4.45*
*p*-value	0.001		0.001		0.001		0.001		0.001	

The table showcases area under receiver operating characteristic curve (ROC-AUC) percentages for each individual label across datasets: VinDr-CXR, ChestX-ray14, CheXpert, UKA-CXR, and PadChest. These datasets were pretrained using SSL on non-medical images (DINOv2) and fully supervised learning on a dedicated chest radiograph dataset (MIMIC-CXR). The total fine-tuning training images for VinDr-CXR, ChestX-ray14, CheXpert, UKA-CXR, and PadChest were *n* = 15,000, *n* = 86,524, *n* = 128,356, *n* = 153,537, and *n* = 88,480, respectively, with corresponding test images totals of *n* = 3,000, *n* = 25,596, *n* = 39,824, *n* = 39,824, and *n* = 22,045, respectively. *p*-values signify the comparison between the average ROC-AUCs from DINOv2 and MIMIC-CXR. For details about each dataset’s labels, refer to Table 3

*N/A* Not available

Together, these results show that both approaches (i) and (ii) have their merits in different regimes: (i) can help to steer the network in the right direction when only few data are available for the training stage, while (ii) prevails when enough training images are available such that fine-tuning of the pretrained weights can be performed on an unrelated task.

## Discussion

We investigated different pretraining methods for the task of image classification in thoracic radiographs. Since AI performance is often dependent on the training and testing domain, we gathered over 800,000 publicly available chest radiographs spanning six distinct institutions across the USA, Europe, and Asia to test our results over a wide variety of different data sources.

Our primary exploration centered around gaining an understanding of the effectiveness and benefits of SSL on non-medical images for the follow-up task of image classification on chest radiographs. We compared three different pretraining strategies: SSL pretraining on a dataset of non-medical images (DINOv2 [18]), supervised pretraining on non-medical images (ImageNet-21 K [13]), and supervised pretraining on medical images (MIMIC-CXR [24]). We employed a state-of-the-art vision transformer [8] architecture and found that SSL on non-medical images serves as a highly effective method for initializing network weights that significantly and consistently improve the ROC-AUC of AI models for chest radiograph classification. Notably, our results demonstrate that under specific circumstances, initializing networks with weights obtained via SSL from non-medical images such as the LVD-142 M dataset [18] can outperform initialization with weights derived from supervised learning on a task-specific, large-scale chest radiograph dataset. This research opens up new perspectives in the application of AI within the medical image analysis domain and has particular importance for situations where large, modality-specific public datasets for pretraining are not available.

The significantly superior performance of models pretrained with SSL on non-medical images based on the DINOv2 [18] method, compared to those pretrained with supervised learning on the ImageNet-21 K [13] dataset, substantiates the claim that weights derived from SSL with non-medical images might better generalize to non-related tasks than weights derived from SL on non-medical images.

It is important to note that these findings were consistent across a variety of imaging findings and across datasets of different origins covering over 800,000 images.

Interestingly, even when compared to supervised learning with a dedicated and the largest public chest radiograph dataset (MIMIC-CXR [24]) to date, the pretraining with SSL on non-medical images demonstrated competitive performance. These results hold promising implications, especially when access to large amounts of annotated medical data is a challenge. Hence, leveraging SSL on non-medical images can be an effective strategy to compensate for the scarcity of annotated medical datasets.

Our study, while yielding promising outcomes for SSL application with non-medical images in medical imagery interpretation, is not without constraints, suggesting avenues for prospective research. Firstly, despite our paired comparison design, we fine-tuned all models with radiograph inputs sized 224 × 224. However, prior studies [59, 60] employing convolutional networks have determined resolutions between 256 × 256 and 448 × 448 to be ample for diagnostic purposes in chest radiographs. Moreover, our chosen network architecture, the ViT [8], has consistently delivered competitive results in existing literature [61–63] with 224 × 224 inputs. Secondly, we propose to extend the analysis to other medical imaging modalities, such as magnetic resonance imaging, computed tomography, or gigapixel imaging in histopathology [64], and for further downstream tasks such as segmentation [65]. Our current endeavor serves as a starting point for exploration into leveraging freely available non-medical images via SSL for medical diagnostics. Third, given the multimodal nature of medical imaging [63], leveraging SSL for these different medical imaging types could yield even richer and more diverse representations, potentially enhancing the diagnostic capabilities of AI models. A persistent challenge, however, remains in sourcing vast volumes of medical images, even if they are unlabeled. Collaborative efforts might be the key to addressing data accessibility challenges.

Our findings highlight the potential of SSL on non-medical images for network initialization in the task of chest radiograph interpretation. The promising results of this approach could inspire further exploration of SSL strategies in the realm of medical imaging, particularly when access to large, annotated medical datasets is limited.

### Supplementary Information

**Additional file 1:Supplementary Table S1.** Performance comparison of the ViT model for label-specific diagnosis on the VinDr-CXR dataset. **Supplementary Table S2.** Performance comparison of the ViT model for label-specific diagnosis on the ChestX-ray14 dataset. The models were pre-trained using self-supervision on natural images (DINOv2) and fully supervised on natural images (ImageNet-21K). Evaluation metrics encompass ROC-AUC, accuracy, sensitivity, and specificity percentages for each label. The ChestX-ray14 dataset comprised *n =* 86,524 fine-tuning training images and *n =* 25,596 test images. 'Healthy' denotes instances where no disease was diagnosed. **Supplementary Table S3.** Performance comparison of the ViT model for label-specific diagnosis on the CheXpert dataset. The models were pre-trained using self-supervision on natural images (DINOv2) and fully supervised on natural images (ImageNet-21K). Evaluation metrics encompass ROC-AUC, accuracy, sensitivity, and specificity percentages for each label. The CheXpert dataset comprised *n =* 128,356 fine-tuning training images and *n =* 39,824 test images. 'Healthy' denotes instances where no disease was diagnosed. **Supplementary Table S4.** Performance comparison of the ViT model for label-specific diagnosis on the MIMIC-CXR dataset. **Supplementary Table S5.** Performance comparison of the ViT model for label-specific diagnosis on the UKA-CXR dataset. The models were pre-trained using self-supervision on natural images (DINOv2) and fully supervised on natural images (ImageNet-21K). Evaluation metrics encompass ROC-AUC, accuracy, sensitivity, and specificity percentages for each label. The UKA-CXR dataset comprised *n =* 153,537 fine-tuning training images and *n =* 39,824 test images. 'Healthy' denotes instances where no disease was diagnosed. **Supplementary Table S6.** Performance comparison of the ViT model for label-specific diagnosis on the PadChest dataset.

## Data Availability

Chest radiograph datasets: ChestX-ray14 and PadChest datasets are publicly available via https://www.v7labs.com/open-datasets/chestx-ray14 and https://bimcv.cipf.es/bimcv-projects/padchest/, respectively. The VinDr-CXR and MIMIC-CXR datasets are restricted-access resources, which can be accessed from PhysioNet under https://physionet.org/content/vindr-cxr/1.0.0/ and https://physionet.org/content/mimic-cxr-jpg/2.0.0/, respectively. CheXpert data may be requested from Stanford University at https://stanfordmlgroup.github.io/competitions/chexpert/. The UKA-CXR dataset contains patient data from the University Hospital Aachen, Germany, and is not yet publicly accessible but can be accessed upon reasonable request to the corresponding authors within a written cooperation agreement. Networks and Python code: The vision transformer network weights, meticulously fine-tuned for chest radiographs, have been archived and are now accessible for research purposes, together with our source code at https://github.com/tayebiarasteh/vit-med. All code for the experiments was developed in Python v3.9 using the PyTorch v2.0 framework. Non-medical datasets and weights: Access to the ImageNet dataset is permitted for academic research purposes from its official site, https://www.image-net.org/, after acceptance of specific terms. Vision transformers that have been pretrained on ImageNet can be accessed for research through the “timm” library at https://github.com/huggingface/pytorch-image-models. DINOv2-based weights for vision transformers, which were pretrained on a compilation of over 142 million images by Meta AI, are available at https://dinov2.metademolab.com/ once specific terms and conditions have been agreed upon.

## Abbreviations

AI: Artificial intelligence
DICOM: Digital Imaging and Communications in Medicine
NLP: Natural language processing
PHI: Protected health information
ROC-AUC: Area under the receiver operating characteristic curve
SL: Supervised learning
SSL: Self-supervised learning
ViT: Vision transformer

## References

[1] Hosny A, Parmar C, Quackenbush J, Schwartz LH, Aerts HJWL (2018). Artificial intelligence in radiology. Nat Rev Cancer.

[2] Müller-Franzes G, Huck L, Tayebi Arasteh S (2023). Using machine learning to reduce the need for contrast agents in breast MRI through synthetic images. Radiology.

[3] Khader F, Han T, Müller-Franzes G (2022). Artificial intelligence for clinical interpretation of bedside chest radiographs. Radiology.

[4] Jaiswal A, Babu AR, Zadeh MZ, Banerjee D, Makedon F (2020). A survey on contrastive self-supervised learning. Technologies.

[5] Krishnan R, Rajpurkar P, Topol EJ (2022). Self-supervised learning in medicine and healthcare. Nat Biomed Eng.

[6] Hendrycks D, Mazeika M, Kadavath S, Song D (2019) Using self-supervised learning can improve model robustness and uncertainty. In: NIPS’19: Proceedings of the 33rd International Conference on Neural Information Processing Systems 15663–15674. https://dl.acm.org/doi/10.5555/3454287.3455690

[7] Vaswani A, Shazeer N, Parmar N, et al. (2017) Attention is all you need. In: NIPS’17: Proceedings of the 31st International Conference on Neural Information Processing Systems 6000–6010. https://dl.acm.org/doi/10.5555/3295222.3295349

[8] Dosovitskiy A, Beyer L, Kolesnikov A, et al. (2021) An image is worth 16x16 words: transformers for image recognition at scale. arXiv. 10.48550/arXiv.2010.11929

[9] Wen Y, Chen L, Deng Y, Zhou C (2021). Rethinking pre-training on medical imaging. J Vis Commun Image Represent.

[10] Krizhevsky A, Sutskever I, Hinton GE (2017). ImageNet classification with deep convolutional neural networks. Commun ACM.

[11] Beyer L, Hénaff OJ, Kolesnikov A, Zhai X, Oord A van den (2020) Are we done with ImageNet? arXiv. 10.48550/arXiv.2006.07159

[12] Ke A, Ellsworth W, Banerjee O, Ng AY, Rajpurkar P (2021) CheXtransfer: performance and parameter efficiency of ImageNet models for chest X-ray interpretation. In: Proceedings of the Conference on Health, Inference, and Learning. Virtual Event USA: ACM 116–124. https://dl.acm.org/doi/10.1145/3450439.3451867

[13] Deng J, Dong W, Socher R, Li LJ, Kai Li, Li Fei-Fei (2009) ImageNet: a large-scale hierarchical image database. In: 2009 IEEE Conference on Computer Vision and Pattern Recognition. Miami, FL, USA 248–255. 10.1109/CVPR.2009.5206848

[14] Krizhevsky A, Hinton G (2009) Learning multiple layers of features from tiny images. University of Toronto, Tech rep. 7. https://www.cs.toronto.edu/~kriz/learning-features-2009-TR.pdf

[15] Everingham M, Van Gool L, Williams CKI, Winn J, Zisserman A (2010). The Pascal Visual Object Classes (VOC) challenge. Int J Comput Vis.

[16] Lin TY, Maire M, Belongie S, et al. (2014) Microsoft COCO: common objects in context. In: Fleet D, Pajdla T, Schiele B, Tuytelaars T, editors. Computer Vision – ECCV 2014. Cham: Springer International Publishing 740–755. (Lecture Notes in Computer Science; vol. 8693). http://link.springer.com/10.1007/978-3-319-10602-1_48

[17] Zhou B, Lapedriza A, Khosla A, Oliva A, Torralba A (2018). Places: a 10 million image database for scene recognition. IEEE Trans Pattern Anal Mach Intell.

[18] Oquab M, Darcet T, Moutakanni T, et al. (2023) DINOv2: learning robust visual features without supervision. arXiv. 10.48550/arXiv.2304.07193

[19] Liu X, Zhang F, Hou Z (2021). Self-supervised learning: generative or contrastive. IEEE Trans Knowl Data Eng.

[20] Azizi S, Mustafa B, Ryan F, et al. (2021) Big self-supervised models advance medical image classification. In: Proceedings of the IEEE/CVF International Conference on Computer Vision (ICCV) 3478–88. https://openaccess.thecvf.com/content/ICCV2021/html/Azizi_Big_Self-Supervised_Models_Advance_Medical_Image_Classification_ICCV_2021_paper.html

[21] Nguyen HQ, Lam K, Le LT (2022). VinDr-CXR: an open dataset of chest X-rays with radiologist’s annotations. Sci Data.

[22] Wang X, Peng Y, Lu L, Lu Z, Bagheri M, Summers RM (2017) ChestX-ray8: hospital-scale chest X-ray database and benchmarks on weakly-supervised classification and localization of common thorax diseases. In: 2017 IEEE Conference on Computer Vision and Pattern Recognition (CVPR) 3462–3471. 10.1109/CVPR.2017.369

[23] Irvin J, Rajpurkar P, Ko M (2019). CheXpert: a large chest radiograph dataset with uncertainty labels and expert comparison. AAAI.

[24] Johnson AEW, Pollard TJ, Berkowitz SJ (2019). MIMIC-CXR, a de-identified publicly available database of chest radiographs with free-text reports. Sci Data.

[25] Tayebi Arasteh S, Isfort P, Saehn M (2023). Collaborative training of medical artificial intelligence models with non-uniform labels. Sci Rep.

[26] Tayebi Arasteh S, Ziller A, Kuhl C, et al. (2023) Private, fair and accurate: training large-scale, privacy-preserving AI models in medical imaging. arXiv. 10.48550/arXiv.2302.01622

[27] Tayebi Arasteh S, Lotfinia M, Nolte T, et al. (2023) Preserving privacy in domain transfer of medical AI models comes at no performance costs: the integral role of differential privacy. arXiv. 10.48550/arXiv.2306.06503

[28] Tayebi Arasteh S, Isfort P, Kuhl C, Nebelung S, Truhn D (2023) Automatic evaluation of chest radiographs – the data source matters, but how much exactly? In: RöFo-Fortschritte auf dem Gebiet der Röntgenstrahlen und der bildgebenden Verfahren. RheinMain CongressCenter (RMCC) in Wiesbaden: Georg Thieme Verlag ab99. 10.1055/s-0043-1763039

[29] Bustos A, Pertusa A, Salinas JM, de la Iglesia-Vayá M (2020). PadChest: a large chest x-ray image dataset with multi-label annotated reports. Med Image Anal.

[30] Isola S, Al Khalili Y (2023) Protected health information. In: StatPearls. Treasure Island (FL): StatPearls Publishing. http://www.ncbi.nlm.nih.gov/books/NBK553131/31985924

[31] Nguyen NT, Truong PT, Ho VT, et al. (2021) VinDr Lab: a data platform for medical AI. https://github.com/vinbigdata-medical/vindr-lab

[32] Shin HC, Roberts K, Lu L, Demner-Fushman D, Yao J, Summers RM (2016) Learning to read chest X-rays: recurrent neural cascade model for automated image annotation. In: Proceedings of the IEEE Conference on Computer Vision and Pattern Recognition (CVPR) 2497–2506. https://openaccess.thecvf.com/content_cvpr_2016/html/Shin_Learning_to_Read_CVPR_2016_paper.html

[33] Leaman R, Khare R, Lu Z (2015). Challenges in clinical natural language processing for automated disorder normalization. J Biomed Inform.

[34] Aronson AR, Lang FM (2010). An overview of MetaMap: historical perspective and recent advances. J Am Med Inform Assoc.

[35] Bird S, Klein E, Loper E (2009) Natural language processing with Python: analyzing text with the natural language toolkit. 1st Edition. Beijing; Cambridge, MA: O’Reilly Media, Inc. 479. https://www.nltk.org/book/

[36] Charniak E, Johnson M (2005) Coarse-to-fine n -best parsing and MaxEnt discriminative reranking. In: Proceedings of the 43rd Annual Meeting on Association for Computational Linguistics - ACL ’05. Ann Arbor, Michigan: Association for Computational Linguistics 173–180. http://portal.acm.org/citation.cfm?doid=1219840.1219862

[37] McClosky D, Charniak E, Johnson M (2010) Automatic domain adaptation for parsing. In: Human Language Technologies: The 2010 Annual Conference of the North American Chapter of the Association for Computational Linguistics [Internet]. Los Angeles, CA, USA: Association for Computational Linguistics 28–36. https://dl.acm.org/doi/abs/10.5555/1857999.1858003

[38] de Marneffe MC, Manning CD (2008) The Stanford typed dependencies representation. In: Coling 2008: Proceedings of the workshop on Cross-Framework and Cross-Domain Parser Evaluation. Manchester, UK: Coling 2008 Organizing Committee 1–8. https://aclanthology.org/W08-1301

[39] Open-i: an open access biomedical search engine. https://openi.nlm.nih.gov

[40] Jaeger S, Candemir S, Antani S, Wáng YXJ, Lu PX, Thoma G (2014). Two public chest X-ray datasets for computer-aided screening of pulmonary diseases. Quant Imaging Med Surg.

[41] Hansell DM, Bankier AA, MacMahon H, McLoud TC, Müller NL, Remy J (2008). Fleischner Society: glossary of terms for thoracic imaging. Radiology.

[42] Peng Y, Wang X, Lu L, Bagheri M, Summers R, Lu Z (2018). NegBio: a high-performance tool for negation and uncertainty detection in radiology reports. AMIA Jt Summits Transl Sci Proc.

[43] de Marneffe MC, Dozat T, Silveira N, et al. (2014) Universal Stanford dependencies: a cross-linguistic typology. In: Proceedings of the Ninth International Conference on Language Resources and Evaluation (LREC’14). European Language Resources Association (ELRA) 4585–4592. https://aclanthology.org/L14-1045/

[44] Caron M, Touvron H, Misra I, et al. (2021) Emerging properties in self-supervised vision transformers. In: Proceedings of the IEEE/CVF International Conference on Computer Vision (ICCV) 9650–9660. https://openaccess.thecvf.com/content/ICCV2021/html/Caron_Emerging_Properties_in_Self-Supervised_Vision_Transformers_ICCV_2021_paper.html

[45] Pizzi E, Roy SD, Ravindra SN, Goyal P, Douze M (2022) A self-supervised descriptor for image copy detection. In: Proceedings of the IEEE/CVF Conference on Computer Vision and Pattern Recognition (CVPR) 14532–14542. https://openaccess.thecvf.com/content/CVPR2022/html/Pizzi_A_Self-Supervised_Descriptor_for_Image_Copy_Detection_CVPR_2022_paper.html

[46] Johnson J, Douze M, Jegou H (2021). Billion-scale similarity search with GPUs. IEEE Trans Big Data.

[47] Zhou J, Wei C, Wang H, et al. (2021) iBOT: image BERT pre-training with online tokenizer. arXiv. 10.48550/arXiv.2111.07832

[48] Caron M, Misra I, Mairal J, Goyal P, Bojanowski P, Joulin A (2020) Unsupervised learning of visual features by contrasting cluster assignments. In: Advances in neural information processing systems 33 9912–9924. https://proceedings.neurips.cc/paper/2020/hash/70feb62b69f16e0238f741fab228fec2-Abstract.html

[49] He K, Fan H, Wu Y, Xie S, Girshick R (2020) Momentum contrast for unsupervised visual representation learning. In: Proceedings of the IEEE/CVF Conference on Computer Vision and Pattern Recognition (CVPR) 9729–9738. https://openaccess.thecvf.com/content_CVPR_2020/html/He_Momentum_Contrast_for_Unsupervised_Visual_Representation_Learning_CVPR_2020_paper.html

[50] Knight PA (2008). The Sinkhorn-Knopp algorithm: convergence and applications. SIAM J Matrix Anal Appl.

[51] Sablayrolles A, Douze M, Schmid C, Jégou H (2019) Spreading vectors for similarity search. In: Proceedings of Proceedings of Seventh International Conference on Learning Representations (ICLR) 2019. https://openreview.net/forum?id=SkGuG2R5tm

[52] Ruan Y, Singh S, Morningstar W, et al. (2023) Weighted ensemble self-supervised learning. In: Proceedings of Eleventh International Conference on Learning Representations (ICLR) 2023. https://openreview.net/forum?id=CL-sVR9pvF

[53] Touvron H, Vedaldi A, Douze M, Jégou H (2019) Fixing the train-test resolution discrepancy. In: Advances in Neural Information Processing Systems 32 (NeurIPS 2019). https://papers.nips.cc/paper_files/paper/2019/hash/d03a857a23b5285736c4d55e0bb067c8-Abstract.html

[54] Loshchilov I, Hutter F (2019) Decoupled weight decay regularization. In: Proceedings of Proceedings of Seventh International Conference on Learning Representations (ICLR) 2019. https://openreview.net/forum?id=Bkg6RiCqY7

[55] Zhou T, Ruan S, Canu S (2019). A review: deep learning for medical image segmentation using multi-modality fusion. Array.

[56] Rezaei-Dastjerdehei MR, Mijani A, Fatemizadeh E (2020) Addressing imbalance in multi-label classification using weighted cross entropy loss function. In: 2020 27th National and 5th International Iranian Conference on Biomedical Engineering (ICBME) 333–338. 10.1109/ICBME51989.2020.9319440

[57] Unal I (2017). Defining an optimal cut-point value in ROC analysis: an alternative approach. Comput Math Methods Med.

[58] Konietschke F, Pauly M (2014). Bootstrapping and permuting paired t-test type statistics. Stat Comput.

[59] Sabottke CF, Spieler BM (2020). The effect of image resolution on deep learning in radiography. Radiol Artif Intell..

[60] Haque MIU, Dubey AK, Danciu I, Justice AC, Ovchinnikova OS, Hinkle JD (2023). Effect of image resolution on automated classification of chest X-rays. J Med Imaging (Bellingham).

[61] He K, Gan C, Li Z (2023). Transformers in medical image analysis. Intelligent Medicine.

[62] Wang B, Li Q, You Z (2023). Self-supervised learning based transformer and convolution hybrid network for one-shot organ segmentation. Neurocomputing.

[63] Khader F, Mueller-Franzes G, Wang T (2023). Multimodal deep learning for integrating chest radiographs and clinical parameters: a case for transformers. Radiology.

[64] Filiot A, Ghermi R, Olivier A, et al. (2023) Scaling self-supervised learning for histopathology with masked image modeling. medRxiv. 10.1101/2023.07.21.23292757

[65] Tayebi Arasteh S, Romanowicz J, Pace DF, et al. (2023) Automated segmentation of 3D cine cardiovascular magnetic resonance imaging. Front Cardiovasc Med 10. 10.3389/fcvm.2023.1167500 10.3389/fcvm.2023.1167500 PMC10613522 37904806

