# Supplementary material for: Enhancing diagnostic deep learning via self-supervised pretraining on large-scale, unlabeled non-medical images
Source: Eur Radiol Exp. 2024 Feb 8;8:10. doi: 10.1186/s41747-023-00411-3 (PMC10850044; doi:10.1186/s41747-023-00411-3)
Supplement: Supplementary file 1 — Additional file 1:Supplementary Table S1. Performance comparison of the ViT model for label-specific diagnosis on the VinDr-CXR dataset. Supplementary Table S2. Performance comparison of the ViT model for label-specific diagnosis on the ChestX-ray14 dataset. The models were pre-trained using self-supervision on natural images (DINOv2) and fully supervised on natural images (ImageNet-21K). Evaluation metrics encompass ROC-AUC, accuracy, sensitivity, and specificity percentages for each label. The ChestX-ray14 dataset comprised n = 86,524 fine-tuning training images and n = 25,596 test images. 'Healthy' denotes instances where no disease was diagnosed. Supplementary Table S3. Performance comparison of the ViT model for label-specific diagnosis on the CheXpert dataset. The models were pre-trained using self-supervision on natural images (DINOv2) and fully supervised on natural images (ImageNet-21K). Evaluation metrics encompass ROC-AUC, accuracy, sensitivity, and specificity percentages for each label. The CheXpert dataset comprised n = 128,356 fine-tuning training images and n = 39,824 test images. 'Healthy' denotes instances where no disease was diagnosed. Supplementary Table S4. Performance comparison of the ViT model for label-specific diagnosis on the MIMIC-CXR dataset. Supplementary Table S5. Performance comparison of the ViT model for label-specific diagnosis on the UKA-CXR dataset. The models were pre-trained using self-supervision on natural images (DINOv2) and fully supervised on natural images (ImageNet-21K). Evaluation metrics encompass ROC-AUC, accuracy, sensitivity, and specificity percentages for each label. The UKA-CXR dataset comprised n = 153,537 fine-tuning training images and n = 39,824 test images. 'Healthy' denotes instances where no disease was diagnosed. Supplementary Table S6. Performance comparison of the ViT model for label-specific diagnosis on the PadChest dataset. [file 41747_2023_411_MOESM1_ESM.docx]

**Enhancing diagnostic deep learning via self-supervised pretraining on large-scale, unlabeled non-medical images**

**ELECTRONIC SUPPLEMENTARY MATERIAL**

**Supplementary Table S1**: Performance comparison of the ViT model for label-specific diagnosis on the VinDr-CXR dataset

| Labels | ROC-AUC | | Accuracy | | Sensitivity | | Specificity | |
| --- | --- | --- | --- | --- | --- | --- | --- | --- |
|  | DINOv2 | ImageNet-21K | DINOv2 | ImageNet-21K | DINOv2 | ImageNet-21K | DINOv2 | ImageNet-21K |
| Cardiomegaly | 94.19 ± 0.58 | 92.99 ± 0.63 | 85.76 ± 1.95 | 83.34 ± 2.62 | 87.16 ± 2.50 | 87.99 ± 2.95 | 85.60 ± 2.36 | 82.80 ± 3.18 |
| Pleural effusion | 97.72 ± 0.62 | 95.47 ± 1.11 | 91.56 ± 2.34 | 91.08 ± 3.26 | 93.44 ± 2.68 | 87.93 ± 3.73 | 91.49 ± 2.49 | 91.20 ± 3.48 |
| Pneumonia | 90.62 ± 1.09 | 90.87 ± 1.04 | 86.12 ± 1.79 | 84.89 ± 2.35 | 83.90 ± 2.84 | 82.78 ± 3.30 | 86.32 ± 2.09 | 85.08 ± 2.75 |
| Atelectasis | 84.56 ± 2.59 | 76.26 ± 2.91 | 79.14 ± 2.75 | 79.37 ± 4.18 | 81.15 ± 4.58 | 65.01 ± 6.17 | 79.08 ± 2.89 | 79.79 ± 4.40 |
| Consolidation | 87.50 ± 2.44 | 91.09 ± 1.69 | 86.27 ± 2.74 | 90.15 ± 2.52 | 81.47 ± 4.61 | 81.16 ± 4.09 | 86.42 ± 2.91 | 90.45 ± 2.65 |
| Pneumothorax | 91.44 ± 4.00 | 88.08 ± 4.92 | 90.47 ± 2.46 | 82.70 ± 4.19 | 78.85 ± 9.13 | 81.83 ± 7.83 | 90.53 ± 2.48 | 82.70 ± 4.21 |
| Pleural thickening | 85.27 ± 1.50 | 82.61 ± 1.72 | 76.79 ± 3.95 | 80.26 ± 6.96 | 79.78 ± 4.60 | 68.91 ± 7.57 | 76.61 ± 4.39 | 80.94 ± 7.77 |
| Lung opacity | 87.20 ± 1.37 | 83.22 ± 1.83 | 71.92 ± 6.39 | 75.75 ± 3.77 | 88.44 ± 5.87 | 79.54 ± 4.88 | 71.44 ± 6.72 | 75.64 ± 3.95 |
| Fibrosis | 84.39 ± 1.49 | 82.67 ± 1.51 | 78.88 ± 3.71 | 77.59 ± 5.93 | 76.99 ± 4.69 | 73.36 ± 6.84 | 79.02 ± 4.29 | 77.92 ± 6.87 |
| Nodule/Mass | 84.77 ± 1.58 | 78.32 ± 1.90 | 75.62 ± 3.94 | 74.63 ± 4.34 | 79.47 ± 4.74 | 72.25 ± 5.38 | 75.38 ± 4.41 | 74.78 ± 4.87 |
| No finding (healthy) | 90.48 ± 0.63 | 88.56 ± 0.69 | 84.91 ± 1.50 | 81.38 ± 1.09 | 88.69 ± 3.59 | 82.73 ± 2.30 | 76.73 ± 3.59 | 78.47 ± 2.45 |
| *Average* | *88.92 ± 4.59* | *86.38 ± 6.27* | *82.49 ± 6.92* | *81.92 ± 6.50* | *83.58 ± 6.93* | *78.50 ± 8.97* | *81.69 ± 7.37* | *81.80 ± 6.88* |

The models were pre-trained using self-supervision on natural images (DINOv2) and fully supervised on natural images (ImageNet-21K). Evaluation metrics encompass the area under the receiver operating characteristic curve (ROC-AUC), accuracy, sensitivity, and specificity percentages for each label. The dataset incorporated n = 15,000 fine-tuning training and n = 3,000 test images. The 'Healthy' label indicates cases with no disease diagnosis.

**Supplementary Table S2**: Performance comparison of the ViT model for label-specific diagnosis on the ChestX-ray14 dataset

| Labels | ROC-AUC | | Accuracy | | Sensitivity | | Specificity | |
| --- | --- | --- | --- | --- | --- | --- | --- | --- |
|  | DINOv2 | ImageNet-21K | DINOv2 | ImageNet-21K | DINOv2 | ImageNet-21K | DINOv2 | ImageNet-21K |
| Cardiomegaly | 88.24 ± 0.48 | 87.58 ± 0.51 | 81.37 ± 1.80 | 79.11 ± 2.45 | 78.72 ± 2.08 | 79.98 ± 2.74 | 81.49 ± 1.95 | 79.08 ± 2.66 |
| Pleural effusion | 81.01 ± 0.33 | 80.73 ± 0.33 | 71.57 ± 1.06 | 71.10 ± 1.60 | 76.87 ± 1.68 | 76.55 ± 2.48 | 70.39 ± 1.63 | 69.89 ± 2.49 |
| Pneumonia | 70.15 ± 1.02 | 69.57 ± 1.05 | 63.01 ± 7.25 | 67.12 ± 4.12 | 66.90 ± 7.31 | 64.24 ± 4.55 | 62.92 ± 7.57 | 67.19 ± 4.30 |
| Atelectasis | 74.71 ± 0.43 | 74.29 ± 0.43 | 68.59 ± 2.28 | 64.99 ± 2.22 | 68.05 ± 2.99 | 71.76 ± 2.98 | 68.67 ± 3.03 | 64.00 ± 2.97 |
| Consolidation | 73.87 ± 0.55 | 73.27 ± 0.55 | 59.95 ± 1.74 | 61.32 ± 2.79 | 79.17 ± 2.09 | 75.96 ± 3.21 | 58.48 ± 2.01 | 60.20 ± 3.23 |
| Pneumothorax | 85.10 ± 0.38 | 85.12 ± 0.38 | 76.10 ± 1.10 | 77.44 ± 1.31 | 80.35 ± 1.51 | 78.40 ± 1.70 | 75.61 ± 1.37 | 77.33 ± 1.64 |
| Fibrosis | 81.26 ± 0.95 | 80.74 ± 0.97 | 74.28 ± 3.71 | 73.01 ± 5.74 | 74.69 ± 3.85 | 74.92 ± 5.85 | 74.27 ± 3.83 | 72.97 ± 5.94 |
| Emphysema | 89.76 ± 0.51 | 86.90 ± 0.56 | 83.96 ± 1.14 | 78.55 ± 1.85 | 81.42 ± 1.55 | 81.53 ± 2.27 | 84.08 ± 1.24 | 78.42 ± 2.02 |
| Hernia | 89.66 ± 1.87 | 88.45 ± 1.89 | 82.06 ± 3.39 | 83.08 ± 7.31 | 82.12 ± 4.72 | 77.55 ± 6.88 | 82.06 ± 3.41 | 83.10 ± 7.35 |
| Pleural thickening | 75.00 ± 0.71 | 74.92 ± 0.71 | 65.73 ± 3.49 | 63.73 ± 3.94 | 72.82 ± 3.81 | 73.53 ± 4.27 | 65.40 ± 3.83 | 63.28 ± 4.31 |
| Edema | 83.34 ± 0.61 | 83.79 ± 0.60 | 73.19 ± 4.30 | 74.19 ± 3.33 | 79.36 ± 4.40 | 80.04 ± 3.49 | 72.96 ± 4.62 | 73.97 ± 3.58 |
| Nodule | 72.24 ± 0.66 | 71.72 ± 0.66 | 74.22 ± 2.18 | 65.62 ± 2.73 | 57.38 ± 2.71 | 66.20 ± 3.25 | 75.36 ± 2.49 | 65.58 ± 3.12 |
| Mass | 79.72 ± 0.58 | 78.20 ± 0.58 | 74.80 ± 2.24 | 74.45 ± 4.83 | 70.37 ± 2.75 | 66.92 ± 5.42 | 75.13 ± 2.59 | 75.00 ± 5.58 |
| No finding (healthy) | 73.00 ± 0.34 | 72.12 ± 0.33 | 70.43 ± 0.59 | 69.99 ± 0.53 | 55.69 ± 2.48 | 55.03 ± 1.98 | 79.67 ± 2.38 | 79.37 ± 1.96 |
| *Average* | *79.79 ± 6.55* | *79.10 ± 6.34* | *72.81 ± 7.43* | *71.69 ± 7.29* | *73.14 ± 8.94* | *73.04 ± 8.23* | *73.32 ± 8.00* | *72.10 ± 7.94* |

The models were pre-trained using self-supervision on natural images (DINOv2) and fully supervised on natural images (ImageNet-21K). Evaluation metrics encompass ROC-AUC, accuracy, sensitivity, and specificity percentages for each label. The ChestX-ray14 dataset comprised n = 86,524 fine-tuning training images and n = 25,596 test images. 'Healthy' denotes instances where no disease was diagnosed.

**Supplementary Table S3**: Performance comparison of the ViT model for label-specific diagnosis on the CheXpert dataset

| Labels | ROC-AUC | | Accuracy | | Sensitivity | | Specificity | |
| --- | --- | --- | --- | --- | --- | --- | --- | --- |
|  | DINOv2 | ImageNet-21K | DINOv2 | ImageNet-21K | DINOv2 | ImageNet-21K | DINOv2 | ImageNet-21K |
| Cardiomegaly | 87.97 ± 0.30 | 87.07 ± 0.31 | 81.70 ± 1.20 | 80.07 ± 1.22 | 79.60 ± 1.60 | 79.58 ± 1.69 | 82.03 ± 1.61 | 80.15 ± 1.64 |
| Pleural effusion | 87.82 ± 0.20 | 87.59 ± 0.20 | 79.17 ± 0.50 | 79.34 ± 0.32 | 83.96 ± 1.94 | 82.39 ± 1.01 | 76.11 ± 1.97 | 77.38 ± 1.01 |
| Pneumonia | 76.33 ± 0.90 | 77.59 ± 0.90 | 73.57 ± 2.98 | 75.55 ± 5.91 | 65.77 ± 3.41 | 65.16 ± 6.06 | 73.79 ± 3.15 | 75.85 ± 6.25 |
| Atelectasis | 69.58 ± 0.41 | 69.25 ± 0.41 | 58.00 ± 3.49 | 57.71 ± 2.71 | 73.04 ± 5.06 | 72.66 ± 3.80 | 55.26 ± 5.04 | 54.98 ± 3.88 |
| Consolidation | 75.12 ± 0.57 | 74.09 ± 0.58 | 61.00 ± 1.53 | 61.94 ± 4.08 | 77.99 ± 1.82 | 74.68 ± 4.58 | 59.90 ± 1.72 | 61.11 ± 4.63 |
| Pneumothorax | 87.25 ± 0.33 | 85.92 ± 0.35 | 79.78 ± 1.29 | 79.03 ± 0.99 | 79.33 ± 1.71 | 77.95 ± 1.35 | 79.84 ± 1.64 | 79.16 ± 1.24 |
| Lung opacity | 73.99 ± 0.29 | 73.78 ± 0.29 | 66.67 ± 0.37 | 67.18 ± 0.43 | 76.94 ± 2.06 | 74.14 ± 2.54 | 58.63 ± 2.06 | 61.73 ± 2.59 |
| Lung lesion | 76.57 ± 0.71 | 75.89 ± 0.72 | 69.87 ± 5.16 | 66.26 ± 3.52 | 69.84 ± 5.50 | 71.12 ± 4.00 | 69.87 ± 5.56 | 66.07 ± 3.80 |
| Fracture | 77.95 ± 0.68 | 76.85 ± 0.69 | 73.71 ± 1.92 | 66.82 ± 1.85 | 68.87 ± 2.31 | 74.42 ± 2.33 | 73.94 ± 2.10 | 66.46 ± 2.03 |
| No finding (healthy) | 87.63 ± 0.30 | 87.61 ± 0.30 | 80.27 ± 1.27 | 79.74 ± 0.63 | 81.47 ± 1.67 | 82.23 ± 0.92 | 80.10 ± 1.65 | 79.39 ± 0.80 |
| *Average* | *80.02 ± 6.60* | *79.56 ± 6.51* | *72.37 ± 8.29* | *71.36 ± 8.39* | *75.68 ± 6.45* | *75.43 ± 6.00* | *70.95 ± 9.69* | *70.23 ± 9.33* |

The models were pre-trained using self-supervision on natural images (DINOv2) and fully supervised on natural images (ImageNet-21K). Evaluation metrics encompass ROC-AUC, accuracy, sensitivity, and specificity percentages for each label. The CheXpert dataset comprised n = 128,356 fine-tuning training images and n = 39,824 test images. 'Healthy' denotes instances where no disease was diagnosed.

**Supplementary Table S4**: Performance comparison of the ViT model for label-specific diagnosis on the MIMIC-CXR dataset

| Labels | ROC-AUC | | Accuracy | | Sensitivity | | Specificity | |
| --- | --- | --- | --- | --- | --- | --- | --- | --- |
|  | DINOv2 | ImageNet-21K | DINOv2 | ImageNet-21K | DINOv2 | ImageNet-21K | DINOv2 | ImageNet-21K |
| Cardiomegaly | 81.50 ± 0.22 | 81.18 ± 0.22 | 69.39 ± 1.44 | 68.85 ± 1.02 | 81.97 ± 2.36 | 82.50 ± 1.68 | 66.13 ± 2.42 | 65.30 ± 1.71 |
| Pleural effusion | 90.89 ± 0.15 | 90.61 ± 0.15 | 81.78 ± 0.82 | 82.21 ± 0.66 | 85.77 ± 1.44 | 84.62 ± 1.19 | 80.56 ± 1.48 | 81.48 ± 1.20 |
| Pneumonia | 74.12 ± 0.50 | 73.82 ± 0.49 | 71.50 ± 2.85 | 70.35 ± 2.55 | 63.78 ± 3.29 | 64.67 ± 3.01 | 72.04 ± 3.27 | 70.75 ± 2.93 |
| Atelectasis | 82.00 ± 0.22 | 81.44 ± 0.23 | 71.35 ± 0.69 | 71.57 ± 1.50 | 80.82 ± 1.16 | 78.84 ± 2.41 | 69.00 ± 1.11 | 69.76 ± 2.46 |
| Consolidation | 82.38 ± 0.44 | 81.82 ± 0.45 | 67.88 ± 2.61 | 68.78 ± 2.09 | 82.85 ± 2.83 | 81.27 ± 2.21 | 67.26 ± 2.84 | 68.26 ± 2.26 |
| Pneumothorax | 86.89 ± 0.39 | 86.07 ± 0.41 | 76.79 ± 1.53 | 78.59 ± 2.17 | 80.21 ± 1.83 | 76.32 ± 2.37 | 76.63 ± 1.67 | 78.70 ± 2.37 |
| Lung opacity | 76.66 ± 0.26 | 76.14 ± 0.27 | 66.19 ± 0.73 | 66.06 ± 0.84 | 75.43 ± 1.20 | 74.79 ± 1.35 | 64.01 ± 1.15 | 64.00 ± 1.33 |
| Lung lesion | 75.31 ± 0.78 | 74.25 ± 0.75 | 74.12 ± 3.30 | 72.53 ± 3.40 | 64.22 ± 3.75 | 63.65 ± 3.71 | 74.38 ± 3.48 | 72.76 ± 3.58 |
| Fracture | 69.81 ± 0.90 | 68.56 ± 0.92 | 73.10 ± 6.00 | 72.43 ± 4.12 | 55.62 ± 6.32 | 54.89 ± 4.45 | 73.47 ± 6.25 | 72.79 ± 4.29 |
| No finding (healthy) | 85.63 ± 0.18 | 85.27 ± 0.19 | 78.66 ± 0.26 | 78.58 ± 0.26 | 78.01 ± 0.64 | 77.56 ± 0.72 | 79.04 ± 0.64 | 79.17 ± 0.70 |
| *Average* | *80.52 ± 6.17* | *79.92 ± 6.35* | *73.08 ± 5.32* | *73.00 ± 5.37* | *72.25 ± 6.04* | *73.91 ± 9.51* | *74.87 ± 10.01* | *72.30 ± 6.16* |

The models were pre-trained using self-supervision on natural images (DINOv2) and fully supervised on natural images (ImageNet-21K). Evaluation metrics encompass ROC-AUC, accuracy, sensitivity, and specificity percentages for each label. The MIMIC-CXR dataset comprised n = 170,153 fine-tuning training images and n = 43,768 test images. 'Healthy' denotes instances where no disease was diagnosed.

| Labels | ROC-AUC | | Accuracy | | Sensitivity | | Specificity | |
| --- | --- | --- | --- | --- | --- | --- | --- | --- |
|  | DINOv2 | ImageNet-21K | DINOv2 | ImageNet-21K | DINOv2 | ImageNet-21K | DINOv2 | ImageNet-21K |
| Cardiomegaly | 85.45 ± 0.18 | 84.86 ± 0.18 | 76.66 ± 0.26 | 76.04 ± 0.24 | 79.29 ± 2.41 | 77.25 ± 1.68 | 74.36 ± 2.42 | 74.97 ± 1.69 |
| Congestion | 84.34 ± 0.32 | 84.22 ± 0.32 | 74.76 ± 1.52 | 73.04 ± 2.39 | 78.70 ± 1.81 | 80.40 ± 2.80 | 74.41 ± 1.80 | 72.39 ± 2.85 |
| Pleural effusion right | 94.11 ± 0.16 | 94.07 ± 0.17 | 85.18 ± 0.67 | 84.59 ± 0.58 | 89.89 ± 0.84 | 90.17 ± 0.81 | 84.76 ± 0.78 | 84.09 ± 0.68 |
| Pleural effusion left | 92.66 ± 0.23 | 92.35 ± 0.24 | 83.02 ± 1.60 | 84.38 ± 0.86 | 87.74 ± 1.80 | 85.81 ± 1.15 | 82.69 ± 1.83 | 84.28 ± 0.99 |
| Pneumonia right | 93.21 ± 0.17 | 92.83 ± 0.18 | 84.20 ± 1.10 | 83.39 ± 0.95 | 86.30 ± 1.46 | 86.64 ± 1.23 | 83.90 ± 1.44 | 82.94 ± 1.24 |
| Pneumonia left | 93.67 ± 0.19 | 93.38 ± 0.20 | 86.09 ± 0.80 | 84.34 ± 0.81 | 85.95 ± 1.00 | 87.78 ± 1.04 | 86.11 ± 0.96 | 84.00 ± 0.98 |
| Atelectasis right | 89.32 ± 0.24 | 89.19 ± 0.24 | 79.76 ± 1.53 | 79.95 ± 0.63 | 83.24 ± 1.86 | 83.13 ± 0.90 | 79.38 ± 1.88 | 79.60 ± 0.76 |
| Atelectasis left | 88.22 ± 0.28 | 87.89 ± 0.28 | 77.76 ± 1.45 | 76.37 ± 1.31 | 83.14 ± 1.75 | 83.99 ± 1.62 | 77.30 ± 1.71 | 75.72 ± 1.55 |
| No finding (healthy) | 86.73 ± 0.18 | 86.28 ± 0.18 | 78.65 ± 0.41 | 77.36 ± 0.45 | 76.50 ± 1.52 | 78.62 ± 1.82 | 79.99 ± 1.52 | 76.57 ± 1.79 |
| *Average* | *89.74 ± 3.57* | *89.45 ± 3.62* | *80.68 ± 4.00* | *79.94 ± 4.29* | *83.42 ± 4.57* | *83.76 ± 4.37* | *80.32 ± 4.44* | *79.39 ± 4.61* |

**Supplementary Table S5**: Performance comparison of the ViT model for label-specific diagnosis on the UKA-CXR dataset

The models were pre-trained using self-supervision on natural images (DINOv2) and fully supervised on natural images (ImageNet-21K). Evaluation metrics encompass ROC-AUC, accuracy, sensitivity, and specificity percentages for each label. The UKA-CXR dataset comprised n = 153,537 fine-tuning training images and n = 39,824 test images. 'Healthy' denotes instances where no disease was diagnosed.

**Supplementary Table S6**: Performance comparison of the ViT model for label-specific diagnosis on the PadChest dataset

| Labels | ROC-AUC | | Accuracy | | Sensitivity | | Specificity | |
| --- | --- | --- | --- | --- | --- | --- | --- | --- |
|  | DINOv2 | ImageNet-21K | DINOv2 | ImageNet-21K | DINOv2 | ImageNet-21K | DINOv2 | ImageNet-21K |
| Cardiomegaly | 92.39 ± 0.25 | 92.09 ± 0.25 | 82.38 ± 1.39 | 82.83 ± 1.84 | 88.52 ± 1.67 | 86.37 ± 2.17 | 81.78 ± 1.67 | 82.48 ± 2.21 |
| Pleural effusion | 95.68 ± 0.26 | 95.42 ± 0.27 | 89.48 ± 0.91 | 89.68 ± 0.57 | 92.15 ± 1.12 | 91.37 ± 0.92 | 89.30 ± 1.03 | 89.57 ± 0.64 |
| Pneumonia | 85.71 ± 0.60 | 85.04 ± 0.63 | 76.21 ± 3.75 | 77.76 ± 1.40 | 80.87 ± 4.11 | 78.45 ± 1.91 | 75.99 ± 4.11 | 77.73 ± 1.53 |
| Atelectasis | 84.07 ± 0.58 | 81.56 ± 0.63 | 76.71 ± 3.16 | 70.10 ± 2.71 | 76.12 ± 3.53 | 79.23 ± 3.10 | 76.74 ± 3.55 | 69.56 ± 3.04 |
| Consolidation | 89.39 ± 0.87 | 89.33 ± 0.73 | 79.66 ± 2.17 | 79.69 ± 2.25 | 87.55 ± 2.44 | 86.69 ± 2.47 | 79.55 ± 2.22 | 79.59 ± 2.30 |
| Pneumothorax | 86.99 ± 2.22 | 89.55 ± 1.80 | 84.61 ± 3.60 | 84.06 ± 3.61 | 76.14 ± 5.22 | 80.24 ± 4.63 | 84.64 ± 3.62 | 84.08 ± 3.63 |
| Emphysema | 88.35 ± 1.07 | 88.19 ± 1.04 | 85.32 ± 4.12 | 83.40 ± 4.60 | 75.82 ± 4.29 | 77.43 ± 4.71 | 85.43 ± 4.21 | 83.46 ± 4.70 |
| Hernia | 94.56 ± 0.77 | 92.86 ± 0.82 | 94.00 ± 2.07 | 91.25 ± 1.46 | 82.84 ± 2.52 | 81.00 ± 2.34 | 94.17 ± 2.13 | 91.41 ± 1.50 |
| Scoliosis | 89.84 ± 0.47 | 88.63 ± 0.55 | 80.64 ± 1.23 | 80.55 ± 1.51 | 83.82 ± 1.61 | 81.58 ± 1.93 | 80.48 ± 1.36 | 80.49 ± 1.66 |
| Congestion | 89.98 ± 1.15 | 89.24 ± 1.24 | 81.66 ± 3.44 | 82.55 ± 2.18 | 82.99 ± 3.98 | 82.56 ± 3.26 | 81.65 ± 3.48 | 82.55 ± 2.21 |
| Aortic elongation | 90.98 ± 0.29 | 90.57 ± 0.31 | 80.36 ± 2.13 | 80.16 ± 1.83 | 87.25 ± 2.40 | 86.93 ± 2.07 | 79.83 ± 2.47 | 79.64 ± 2.12 |
| Kyphosis | 89.08 ± 0.70 | 89.50 ± 0.68 | 80.73 ± 3.87 | 81.17 ± 2.59 | 82.94 ± 3.92 | 83.53 ± 2.85 | 80.68 ± 4.04 | 81.12 ± 2.70 |
| COPD | 84.33 ± 0.37 | 84.46 ± 0.37 | 73.53 ± 1.93 | 72.20 ± 1.36 | 80.62 ± 2.55 | 83.08 ± 1.84 | 72.45 ± 2.59 | 70.55 ± 1.82 |
| Pleural thickening | 84.20 ± 0.76 | 82.95 ± 0.76 | 72.54 ± 1.98 | 71.80 ± 3.71 | 81.51 ± 2.49 | 79.71 ± 4.05 | 72.26 ± 2.11 | 71.56 ± 3.94 |
| Nodule/Mass | 74.99 ± 0.83 | 74.29 ± 0.83 | 72.07 ± 3.66 | 64.05 ± 4.97 | 63.99 ± 4.05 | 72.18 ± 5.31 | 72.44 ± 3.99 | 63.69 ± 5.43 |
| Infiltrates | 82.62 ± 0.64 | 81.30 ± 0.66 | 70.07 ± 1.83 | 70.14 ± 3.60 | 82.60 ± 2.27 | 79.14 ± 3.86 | 69.54 ± 1.99 | 69.75 ± 3.91 |
| No finding (healthy) | 86.43 ± 0.25 | 86.09 ± 0.24 | 77.05 ± 0.65 | 77.04 ± 0.36 | 82.51 ± 1.68 | 81.07 ± 0.80 | 74.40 ± 1.69 | 75.09 ± 0.77 |
| *Average* | 87.62 ± 4.86 | *87.12 ± 5.05* | *79.82 ± 6.69* | *78.73 ± 7.49* | *81.66 ± 6.91* | *81.80 ± 5.30* | *79.49 ± 6.97* | *78.37 ± 7.80* |

The models were pre-trained using self-supervision on natural images (DINOv2) and fully supervised on natural images (ImageNet-21K). Evaluation metrics encompass ROC-AUC, accuracy, sensitivity, and specificity percentages for each label. The PadChest dataset comprised n = 88,480 fine-tuning training and n = 22,045 test images. 'Healthy' denotes instances where no disease was diagnosed. *COPD* Chronic obstructive pulmonary disease.
